# Harmonic dependence of thermal magnetic particle imaging

**DOI:** 10.1038/s41598-023-42620-1

**Published:** 2023-09-22

**Authors:** Thinh Q. Bui, Mark-Alexander Henn, Weston L. Tew, Megan A. Catterton, Solomon I. Woods

**Affiliations:** 1 National Institute of Standards and Technology, Gaithersburg, MD 20895 USA; 2 Department of Chemistry and Biochemistry, University of Maryland, College Park, MD 20742 USA; 3 Department of Mechanical Engineering, Johns Hopkins University, Baltimore, MD 21287 USA

**Keywords:** 3-D reconstruction, Imaging techniques, Nanoparticles, Imaging techniques, Magnetic properties and materials, Biomedical engineering

## Abstract

Advances in instrumentation and tracer materials are still required to enable sensitive, accurate, and localized in situ 3D temperature monitoring by magnetic particle imaging (MPI). We have developed a high-resolution magnetic particle imaging instrument and implemented a low-noise multi-harmonic lock-in detection method to observe and quantify temperature variations in iron oxide nanoparticle tracers using the harmonic ratio method for determining temperature. Using isolated harmonics for MPI and temperature imaging revealed an apparent dependence of imaging resolution on harmonic number. Thus, we present experimental and simulation studies to quantify the imaging resolution dependence on temperature and harmonic number, and directly validate the fundamental origin of MPI imaging resolution on harmonic number based on the concept of a harmonic point-spread-function.

## Introduction

Magnetic particle imaging (MPI) is a tracer-based imaging modality that is experiencing rapid growth towards biomedical and clinical applications^1–15^. In this work, our efforts are directed at establishing magnetic nanoparticles (MNP) as a traceable nano-thermometer standard for *in situ* accurate temperature sensing and imaging, relying on MPI as a core technology. Since MPI provides a non-invasive, image-guided approach towards heat-based theranostics, numerous authors have discussed potential prospects for MPI-based temperature sensing^16–20^ and hyperthermia^21–26^ to complement the capabilities of ultrasound, fluoroscope, computed tomography (CT) and magnetic resonance imaging (MRI)^27,28^. Achieving accurate MPI-based thermal imaging and targeted heat-based therapy is demanding and requires a systematic evaluation of the effects of technical noise and temperature on detection sensitivity and imaging resolution.

While most MPI studies have focused on measurements of in situ concentration distributions of superparamagnetic tracers, nanoparticle thermometry has also been a longstanding goal of MPI^18,26^. Two key approaches for nanoparticle thermometry have been proposed: multi-color, system matrix-based^18,29,30^ and multi-harmonic-based measurements^16,19,20,31^. The system matrix approach requires measurements of calibration system matrices to reconstruct both concentration and temperature, and a linear interpolation step for intermediate temperatures^18^. Generating a fine grid of temperature calibration matrices is not only time-consuming, but achieving high temperature spatial resolution in image reconstruction is difficult if the variation of the particle response with temperature is small^32^. Here, we will focus on the multi-harmonic technique, where the temperature can be determined by measurement of two harmonics and their ratios^16^. The harmonic approach requires only a single calibration matrix of particle response across magnetic field and temperature, which is used for image reconstruction of temperature spatial profiles if the excitation magnetic field is known. This is akin to determining the reference function of resistance-based thermometers, where the analog in our case would be the harmonic response function of the magnetic nanoparticle sensors. By avoiding the interpolation required by the system matrix approach, the harmonic approach in principle could achieve more accurate results with a higher thermal spatial resolution. Even though nanoparticle thermometry based on measurement of individual harmonics has been presented previously^17,19,31,33–36^ the effects of temperature and harmonic number on MPI imaging (sensitivity and spatial resolution) are not quantitatively understood. By both experiments and simulations, we investigate the impact of temperature and harmonic measurement on MPI, which will have important implications for image reconstruction whenever temperature is involved.

For MPI, the magnetization response of MNP is driven into saturation by a sinusoidal excitation field and most often detected by inductive coil sensors. Typical MPI systems record the magnetization response in the time domain with a digitizer. While this procedure has the advantage of detecting a broadband harmonic signal, often exceeding 1 MHz, the broad bandwidth carries along with it excess broadband noise. Broadband noise-matching is a possible solution, but the complex impedance of the inductive coil introduces frequency-dependence of noise that presents complications for bandwidths exceeding 1 MHz^37–42^. Towards sensitive temperature measurements with the multi-harmonic technique, we use an alternative and more general approach by implementing a parallel lock-in detection scheme in which many harmonics are measured simultaneously while still leveraging the narrowband detection and low-noise properties of lock-in amplifiers. In contrast to Goodwill et al.^37^, we are detecting many harmonics from the particle response in a narrowband fashion, thereby overcoming the resolution-bandwidth trade-off. It is a general goal of magnetic imaging to reach the body noise limit, which can potentially be achieved at a high frequency (1 MHz to 100 MHz) by detecting at a high harmonic number and reaching coil resistance (thermal) noise. Here, we present our multi-harmonic lock-in detection approach to compare with the traditional digitizer-based measurement, and provide an in-depth characterization of the harmonic and temperature dependence of MPI.

## Results

### MPI characterization

**Figure 1:**
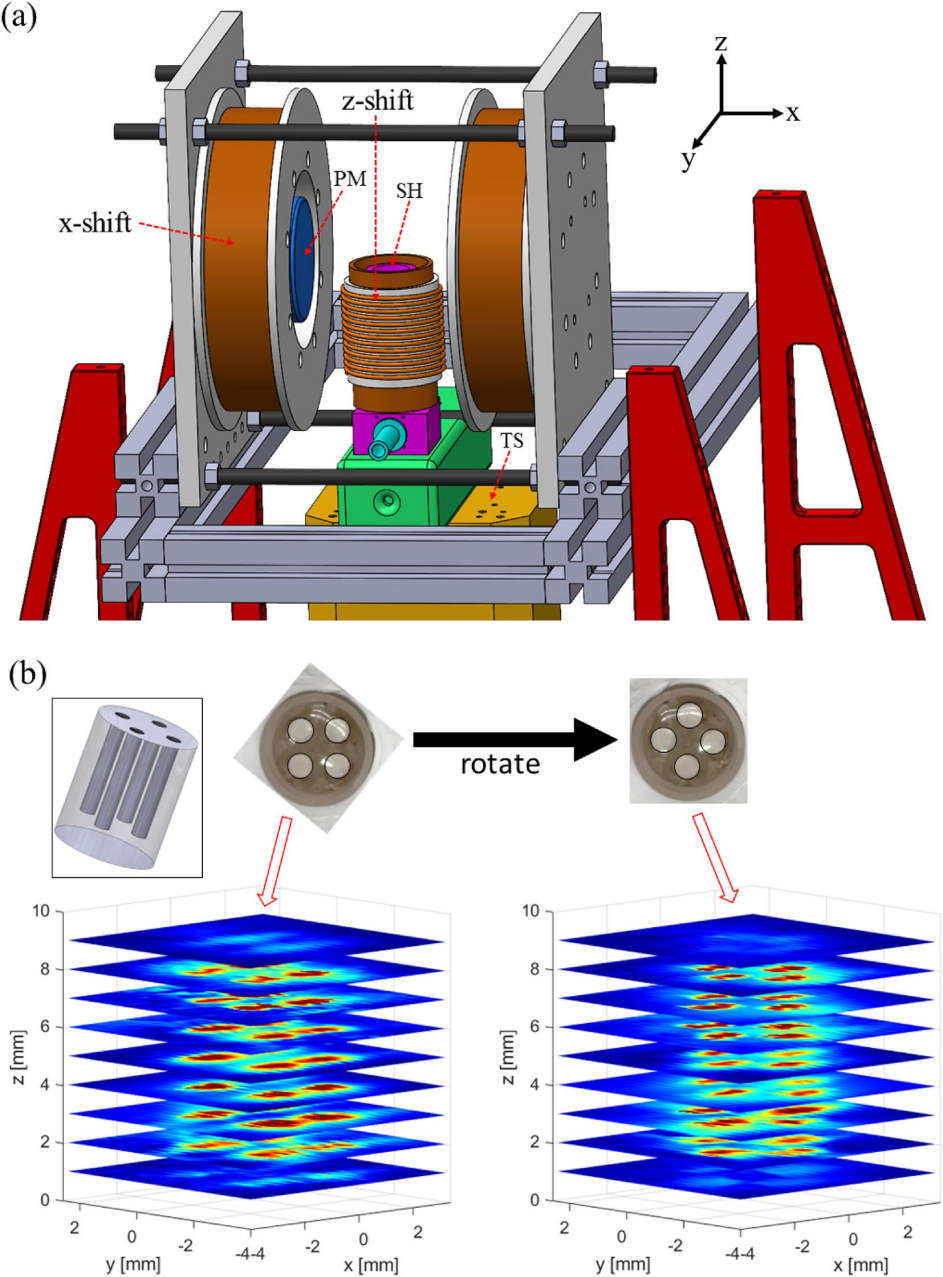
Magnetic particle imaging: (**a**) MPI instrument schematic. PM is the permanent magnet pair to set the gradient field. SH is the sample holder. The excitation coil is concentric to the SH but not visible. Electromagnetic shifting is achieved with labeled shift coils (x, z). Alternatively, 3D mechanical translation is performed with the stages denoted as TS. (**b**) 4-channel glass phantom containing undiluted (5 mg/ml of Fe) Vivotrax tracer at two rotated orientations shown from the top view. The four white circles (diameter = 1 mm, height = 8 mm) are the sample channels. 3D-MPI measurement of the phantom, measured by mechanically scanning voxels in steps of ($0.25\times 0.25\times 1$) mm to map out the 3D phantom image at the two orientations.

**Figure 2:**
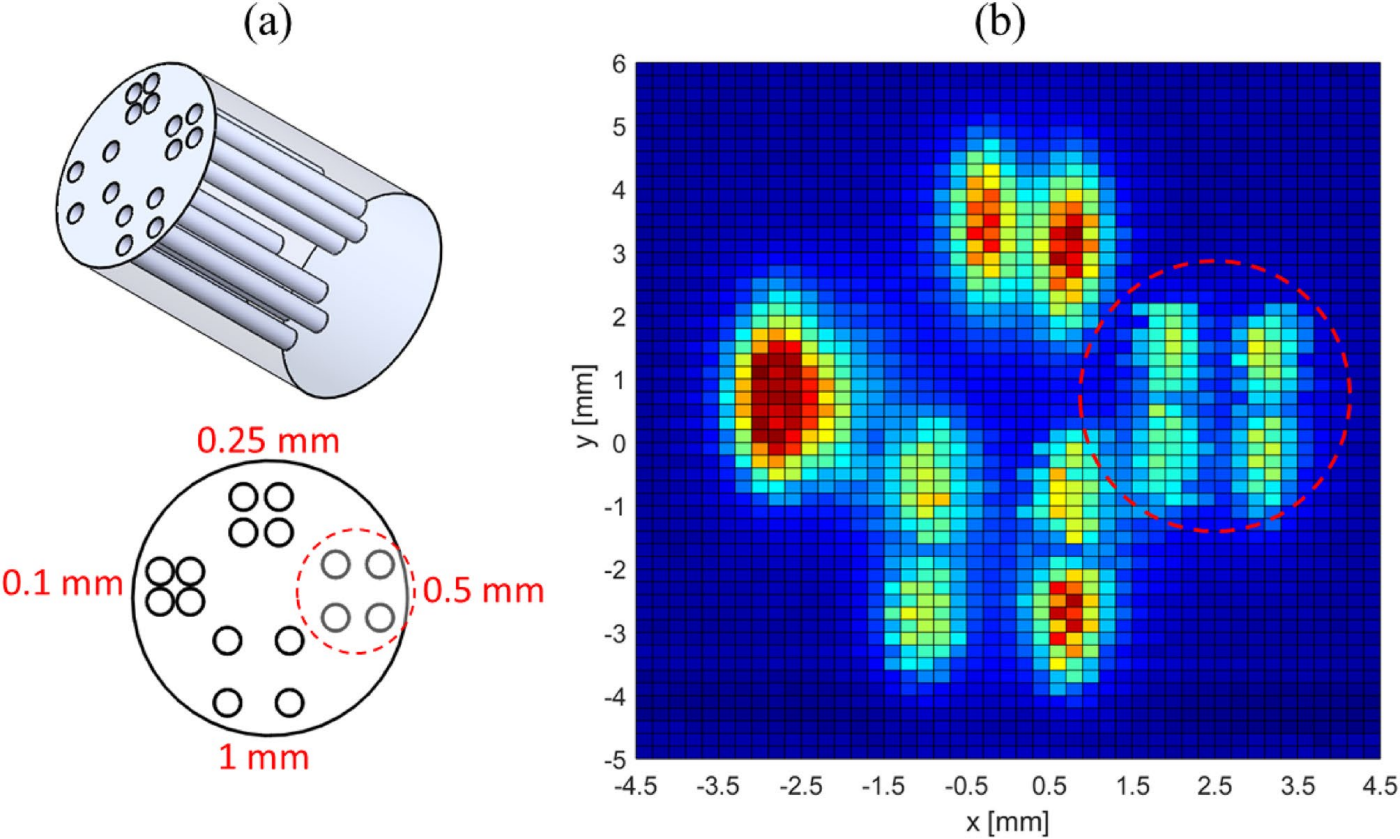
(**a**) 3D-printed acrylic spatial resolution phantom with 16 channels. The diameter of each channel is 0.75 mm. The group of 4 channels have edge-to-edge separations indicated by the labels on the top view cartoon. (**b**) 2D MPI image. For clarity, the dashed red circle denotes the MPI image-to-cartoon correspondence for channels with 0.5 mm separation.

The MPI instrumentation (Fig. 1a, see Methods section for more detail) was designed for quantitative thermal imaging using magnetic nanoparticles. To assess imaging spatial resolution, we use a glass phantom with dimensions of 8 mm x 12 mm (diameter x height). The phantom has four channels (1 mm diameter, 8 mm height) where MNP samples can be introduced and sealed by epoxy. To demonstrate 3D capabilities, Fig. 1b presents 3D MPI images of this glass phantom in two rotated orientations, with four clearly resolved channels. For a more systematic evaluation of spatial resolution limit, we used a 3D printed acrylic phantom shown in Fig. 2. Here, the sets of four channels have different edge-to-edge hole spacing to evaluate the spatial resolution based on the Rayleigh criterion. The 2D image in Fig. 2 reveals that we can resolve 250 μm in the x-direction, the direction of the highest gradient field. Our results are competitive with the highest resolution MPI instruments reported^9,43,44^. These results, along with the measured point-spread-function (PSF^45,46^) of a commercial MNP, Vivotrax, indicate that the magnitude of the gradient field, *G*, is $\approx 20$ T/m for the x-axis and $\approx 10$ T/m for the y-axis. These results are consistent with our COMSOL simulations of the permanent magnet pair. We note that such a high gradient field is challenging to transfer to clinical settings. However, it has been shown^47^ that MPI using super-ferromagnetic (SFM) nanoparticles with their narrow PSF can achieve superior spatial resolution despite a lower gradient field. But for commercial nanoparticles, this work and previous MPI reports^9,43,44^ show that a high gradient field is required to achieve sub-millimeter native spatial resolution.

#### Multi-harmonic detection for MPI

A voltage-based measurement system using an inductive coil (IC) sensor with a pre-amp has SNR given by^37,40^:

1 $$\begin{aligned} SNR \propto \frac{\varepsilon \cdot \frac{dM}{dt}}{\sqrt{(4k_BTR_L+\bar{e}_n^{2}+\bar{i}_n^{2}|Z_L|^{2})\cdot \Delta f}} \end{aligned}$$

The quantity in the numerator is the product of the coil sensitivity, $\varepsilon$, and voltage induced by the MNP’s modulated magnetization, *dM*/*dt*. The denominator denotes the noise sources from the sensor, an IC sensor with real ($R_L$) and complex impedance ($Z_L$), and the pre-amp at temperature *T*. The pre-amp has a voltage and current noise spectral density (NSD, $V/\sqrt{Hz}$), $\bar{e}_n$ and $\bar{i}_n$, respectively. Since $\Delta f$ is the detection bandwidth, the best SNR is obtained by minimizing the bandwidth. This ideal scenario is, however, at odds with the MPI technique in that it is desirable to retain all information-carrying harmonics by using a high sampling rate (2 MSa/s for 1 MHz). Reducing the sampling rate (detection bandwidth) could improve SNR, but at the expense of spatial resolution^45^. To overcome the incompatibility of noise-matching and the bandwidth requirement, we implemented lock-in detection with parallel multi-harmonic capabilities that can improve SNR by permitting detection at arbitrarily high frequencies limited only by the lock-in amplifier bandwidth.

Relying on measurement of only a few harmonics has been proposed for in situ temperature measurement^16,20^, and also as a general modality for imaging^13,42^. Isolated harmonics can be sensitively measured with lock-in detection, where the noise bandwidth (1 Hz to 100 Hz range) is adjustable based on the desired acquisition speed and sensitivity. For detection of a single harmonic, the equivalent noise bandwidth ($\Delta f_{LI}$) is set by the acquisition time, $\tau$, which is typically 10 ms in our experiment. For a first order filter, $\Delta f_{LI} = 1/4\tau$, which gives a $\Delta f_{LI} \approx 25$ Hz. The noise bandwidth of the Zurich lock-in (Zurich, HF2LI, 50 MHz) scales as $\Delta f_{LI} \propto n^{-5/4}$, where *n* is an integer denoting the filter order number. Increasing the filter order is a simple way to lower the $\Delta f_{LI}$ without increasing $\tau$. For the equivalent measurement of harmonic amplitudes with a digitizer, the noise bandwidth is related to the acquisition time $\tau$ by $\Delta f_{D} = F_s/N = 1/\tau$, where $F_s$ is the sampling rate and *N* is the sample length.

**Figure 3:**
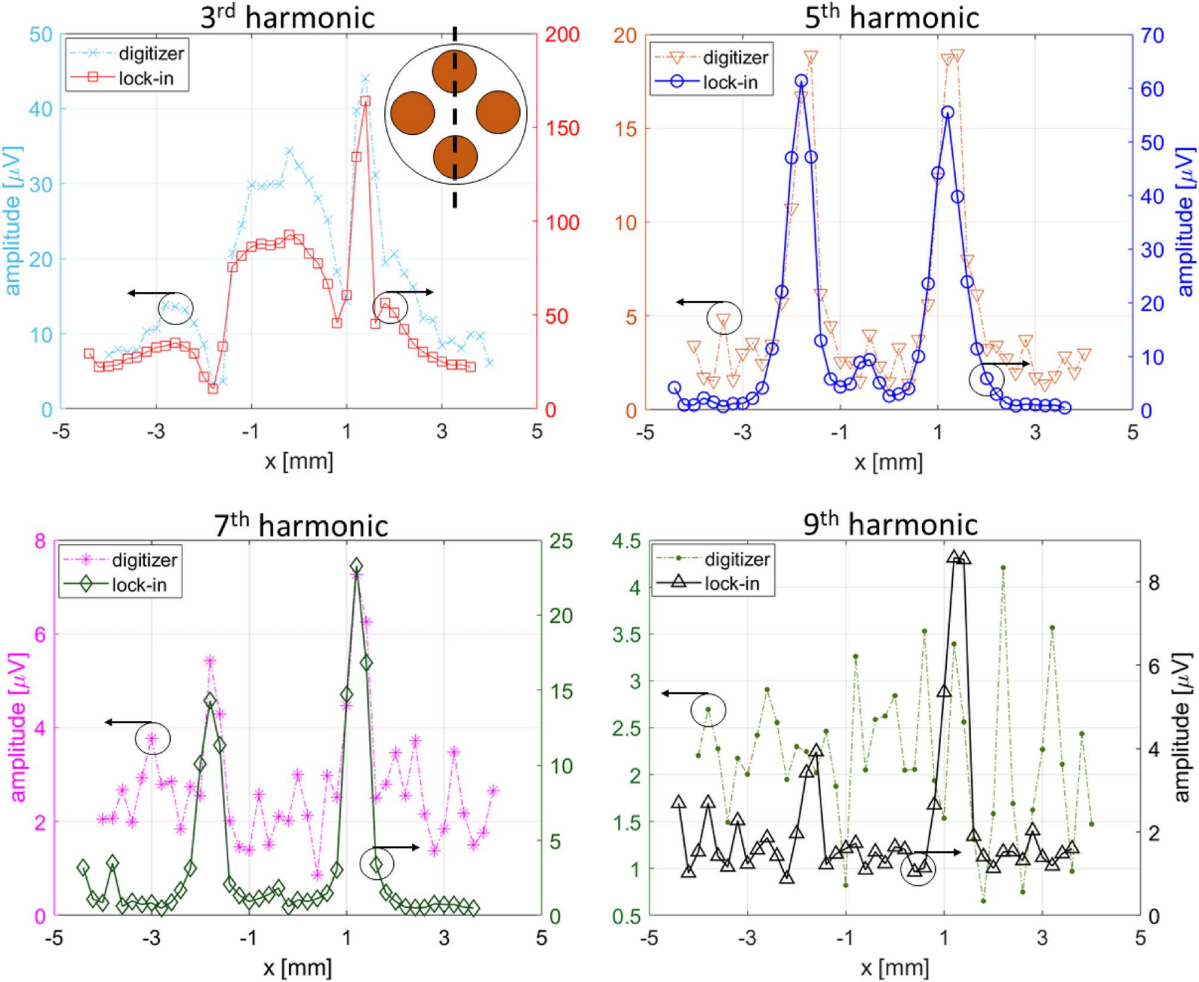
1D scan of Vivotrax tracer in the glass phantom from Fig. 1 measured by a lock-in and digitizer. The two peaks correspond to MNP concentrations in two of the four sample channels as indicated by the cartoon in the 3rd harmonic panel.

To compare the noise performance of multi-harmonic lock-in detection with the digitizer for MPI, we acquired images of the Vivotrax glass phantom using both techniques. Figure 3 show the 1D image slice across two channels of the glass phantom. The four panels show a comparison of the 3rd to 9th harmonic signals measured by the lock-in and digitizer. Six harmonics were recorded simultaneously in the lock-in case, but only four are presented for clarity. Here, the magnetization data was recorded at each spatial position using the same measurement time of $\tau$ = 10 ms for each instrument. This acquisition time corresponds to noise bandwidths of 100 Hz and 25 Hz for the digitizer and lock-in, respectively, for each harmonic. From Eq. (1), the corresponding SNR enhancement is $\sqrt{100/25}=2$. Combined with the lower noise floor of the lock-in relative to the digitizer, the improved SNR (signal peak divided by the RMS noise of the baseline) with the lock-in is most clearly observed in the higher harmonic data of Fig. 3. In general, the lock-in amplifier is not the limiting factor for MPI imaging speed since the integration time can be arbitrarily set as short as 1 $\mu s$. The fundamental limitation in our imaging speed is the slow raster scan mechanisms (mechanical translation or electromagnetic shifting), which is slower than the Lissajous scanning at $\approx$ 25 kHz in each axis. In principle, the lock-in amplifier can be implemented with the Lissajous scanning, as long as the lock-in data acquisition and Lissajous scanning are synchronized.

#### Temperature dependent MPI

Temperature affects the equilibrium magnetization and dynamics of MNP, which are encoded on the MPI signal. For MNP systems that display magnetization dynamics at experimentally-resolvable timescales, we have shown the effect of temperature on distinct Néel and Brownian mechanisms^48^. There have been numerous other reported studies on the temperature dependence of suspended magnetic nanoparticles^16–20,26,31,33–36,49,50^ that focused on determining the magnetization response sensitivity to temperature. Those that rely on harmonic measurements^16,20,31,33,36^, as opposed to the system matrix approach, to measure temperature typically select low harmonics (2nd to 5th) that display the largest signal to improve measurement sensitivity. However, these reports did not provide an assessment of how harmonic measurements impact imaging spatial resolution, a key component for thermal imaging performance. Here, to investigate the impact of temperature and harmonic-based measurements on MPI imaging resolution, we used our high resolution MPI instrument with the imaging phantom in Fig. 1 to characterize these dependencies.

**Figure 4:**
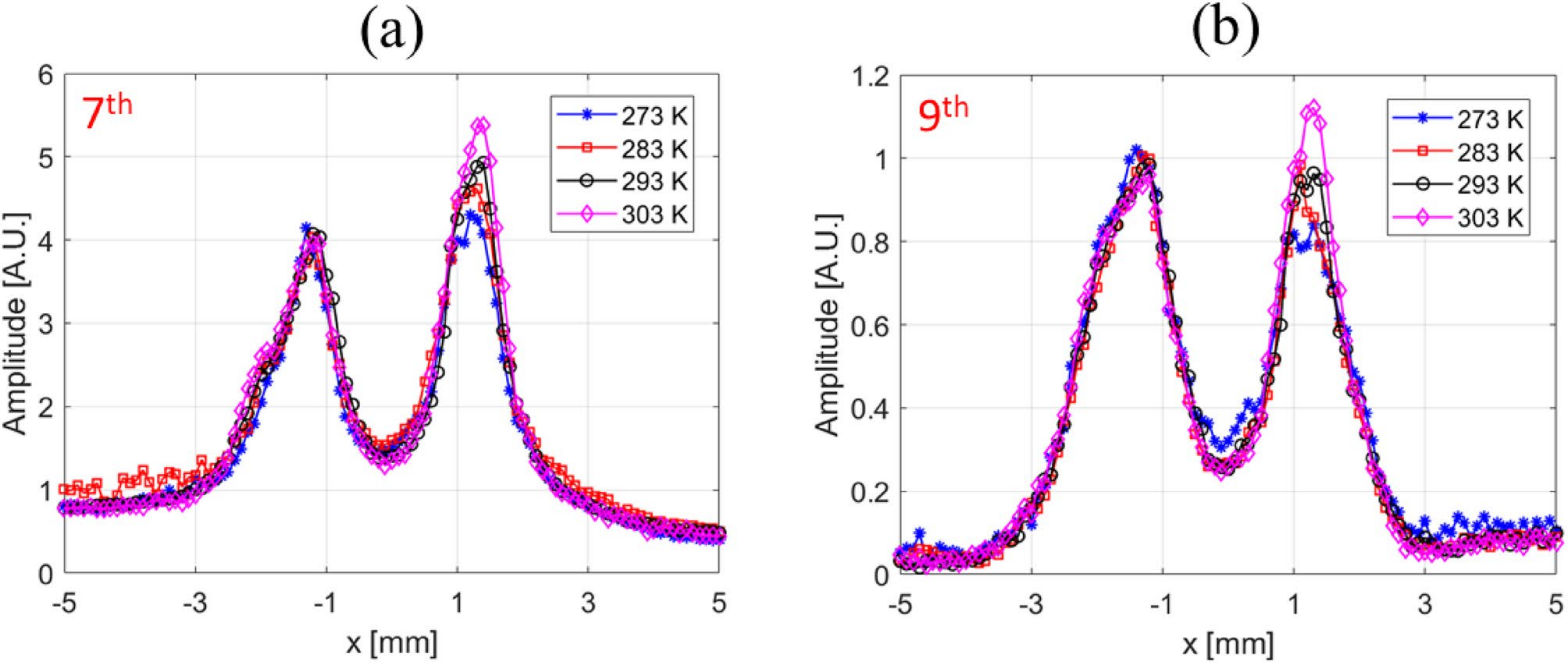
1D images of Vivotrax as a function of temperature for the 7th (**a**) and 9th (**b**) harmonics for the temperature dependence of spatial resolution.

**Figure 5:**
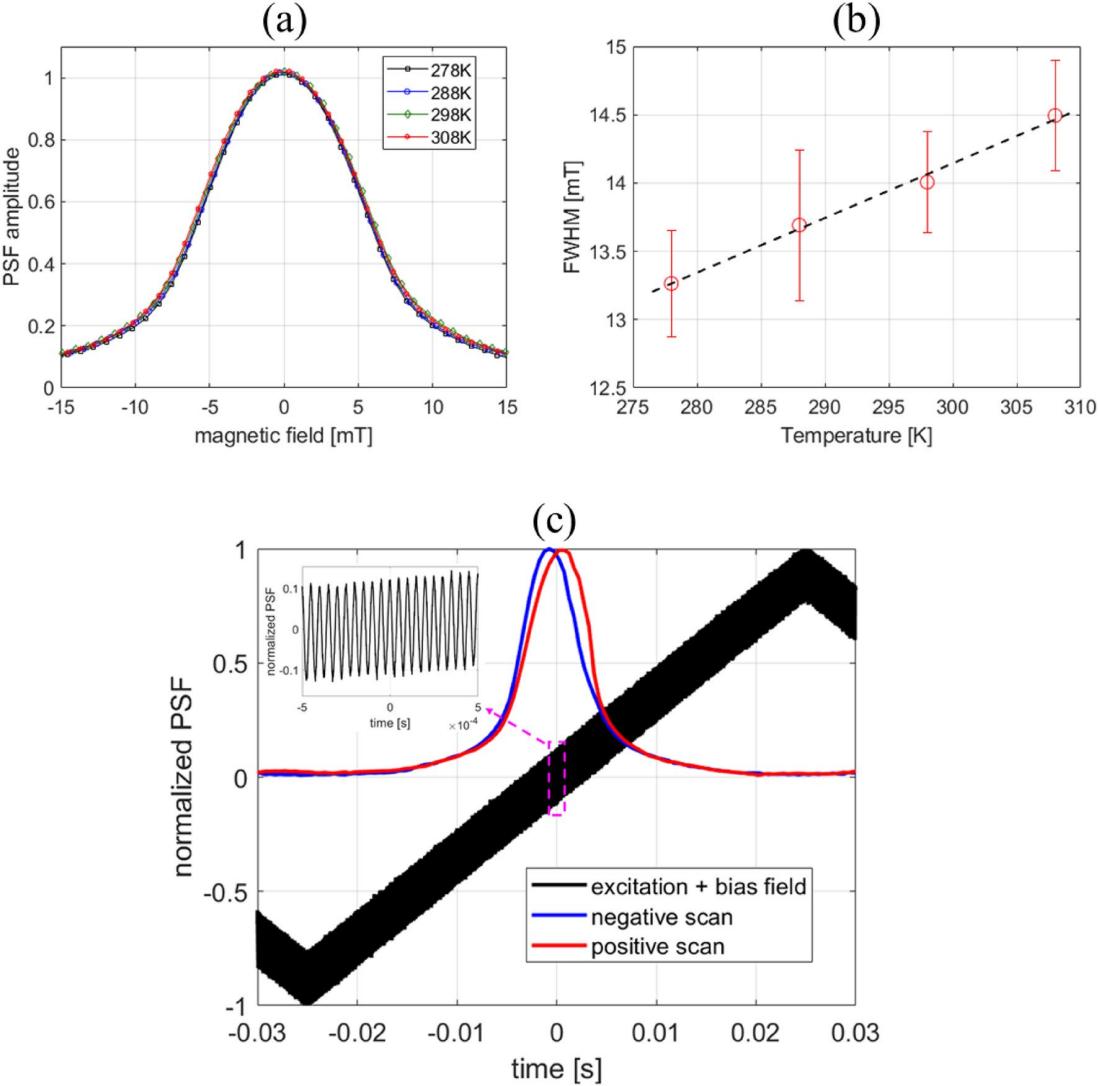
(**a**) Vivotrax PSF and (**b**) fitted FWHM as a function of temperature (slope = (0.041 ±0.006) mT/K). (**c**) Positive and negative scan PSFs for Vivotrax show minimal relaxation effects (asymmetry). The inset shows the high frequency sinusoidal excitation field superimposed on the 10 Hz triangle bias field. The PSF includes information from all harmonics.

Using Vivotrax samples in the glass phantom, we studied the imaging spatial resolution dependence on harmonic number and temperature. 1-D scans across the two channels of the phantom are presented in Fig. 4 for both the 7th and 9th harmonics. Only a modest change in the full-width-half-maximum (FWHM) of the peaks was observed as a function of temperature. A more quantitative assessment of temperature on spatial resolution can be ascertained from PSF data (Fig. 5). In comparison with measurements in Fig. 4, the PSF in Fig. 5 includes all harmonics and therefore exhibits significantly higher SNR. By fitting the PSF curves to a Voigt function, the temperature dependence of the FWHM was obtained (Fig. 5(b)). A modest slope of (0.041±0.006) mT/K is consistent with a small temperature dependence for data presented in Fig. 4. Finally, Fig. 5c shows the PSF for FFP scans in the positive and negative directions for Vivotrax at 298 K. The high symmetry of the PSF for both directions suggests a small relaxation effect at our measurement conditions^51^.

**Figure 6:**
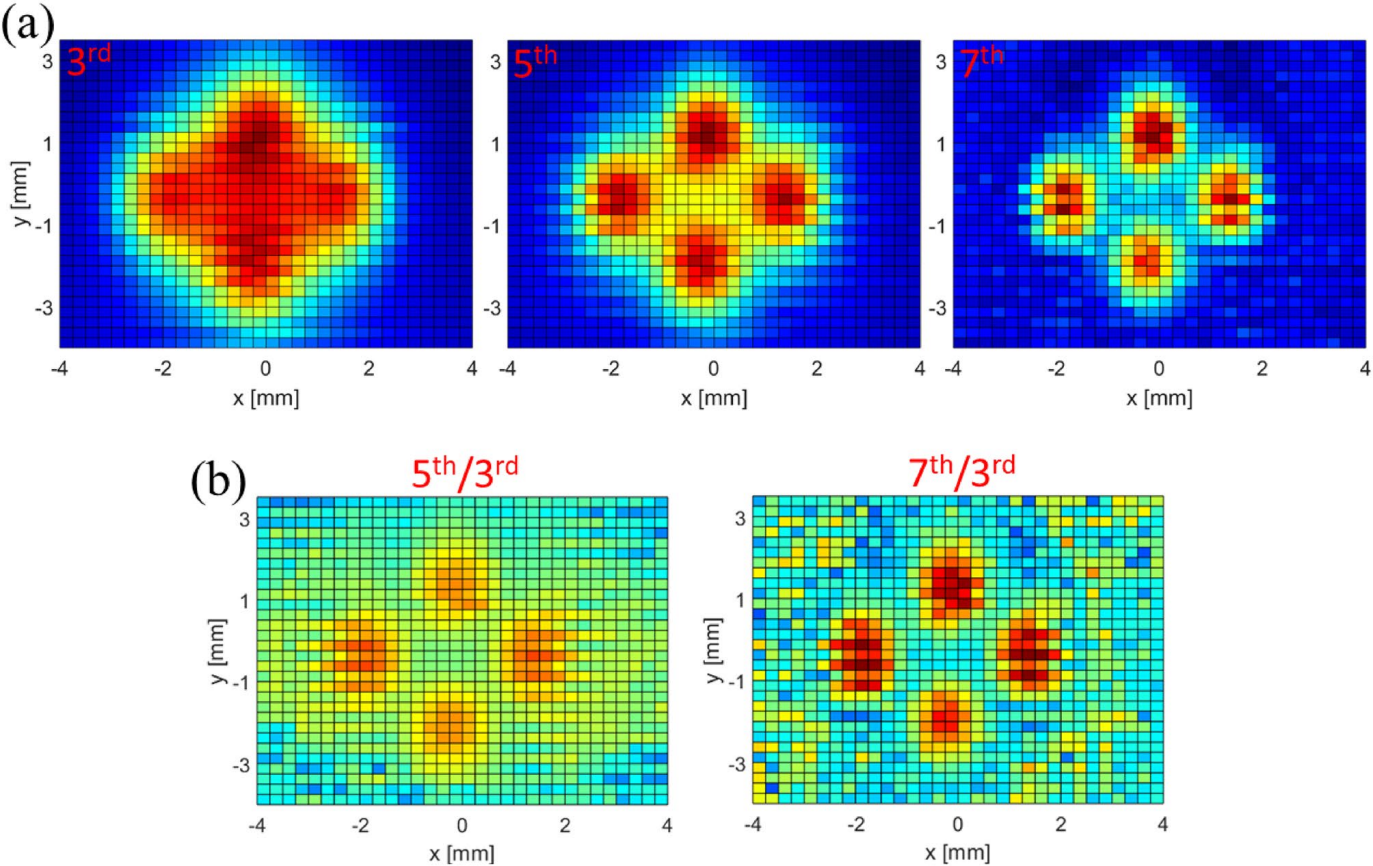
Spatial resolution dependence on (**a**) harmonic number and (**b**) harmonic ratio for 70 nm Synomag nanoparticles.

For thermal imaging applications using magnetic nanoparticles, measurement of harmonic ratios, rather than individual harmonic amplitudes, has been proposed to remove the concentration dependence^16^. We recorded 2D images using individual harmonics and also their ratios, and the data is presented in Fig. 6. In the case of the 5th/3rd and 7th/3rd harmonic ratios, the higher harmonic dominates the spatial resolution of the ratio image, which mostly resembles the image of the higher harmonic number. In general, images obtained with either individual harmonics or harmonic ratios clearly show a harmonic dependence of spatial resolution, and suggests a corresponding PSF dependence on harmonic number. The concept of a harmonic PSF is crucial for this imaging modality, and will be discussed in detail in the following sections.

### Harmonic dependence of spatial resolution

Most MPI instruments typically utilize all harmonics for image reconstruction, which obscures the impact of individual harmonics on spatial resolution. The effect of harmonics on the reconstructed image has been introduced previously^2^, especially in the context of feedthrough (1st harmonic) suppression^52^. More importantly, these studies reveal a direct mapping of harmonic frequency space to spatial frequency in the image space for sinusoidal excitation. Our work extends upon these analyses by experimentally determining the impact of individual harmonics on image reconstruction, a new concept denoted as the harmonic PSF.

**Figure 7:**
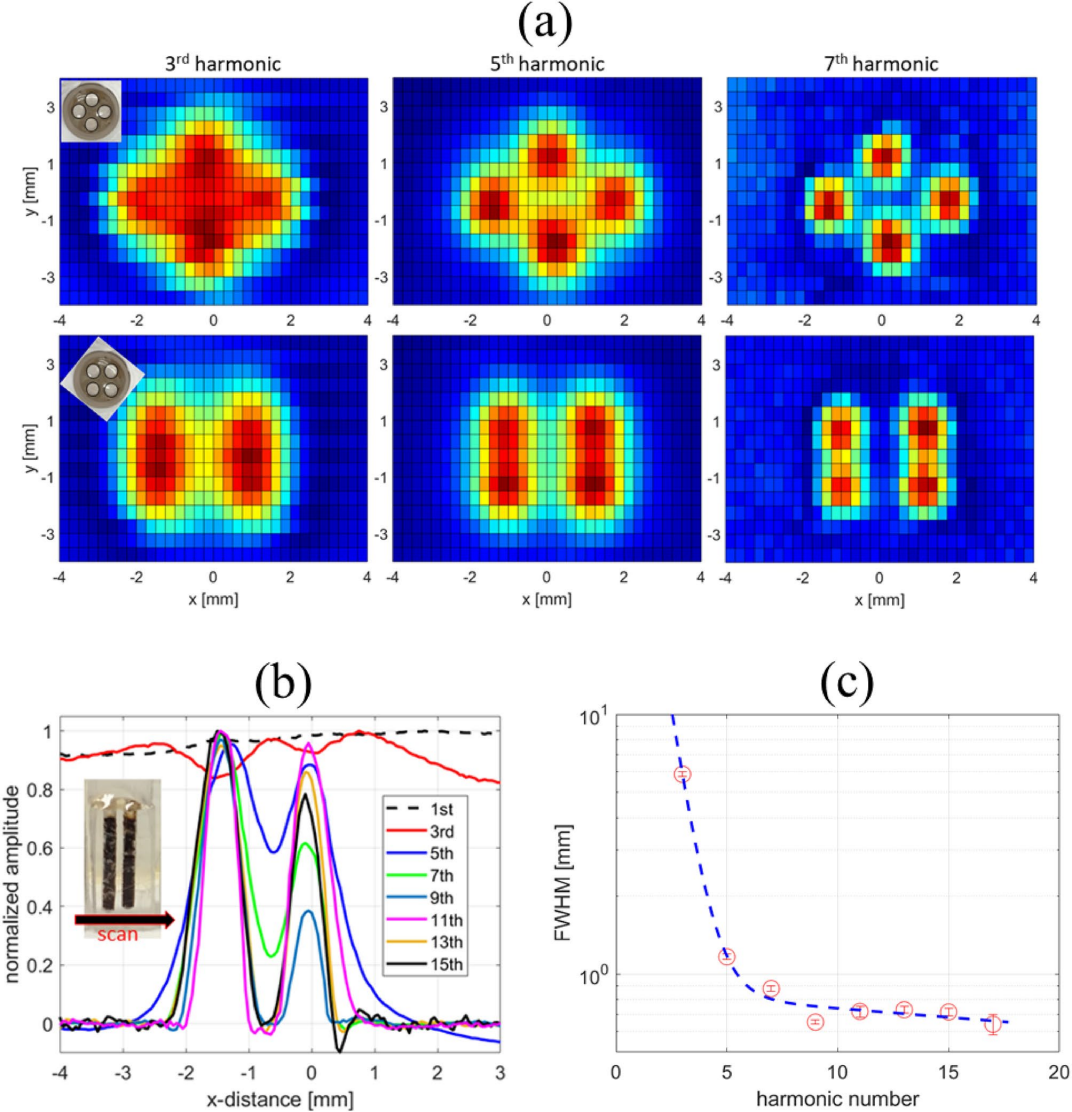
(**a**) 2D-MPI images of undiluted (15 mg/ml) 70 nm Synomag nanoparticles in a glass phantom. The apparent spatial resolution improves with increasing harmonic number. (**b**) An acrylic phantom (inset) is used to obtain the 1D image for quantifying the spatial resolution dependence on harmonic number. (**c**) Fitted FWHM from (**b**) along with a bi-exponential fit (dashed blue curve) to the FWHM.

**Figure 8:**
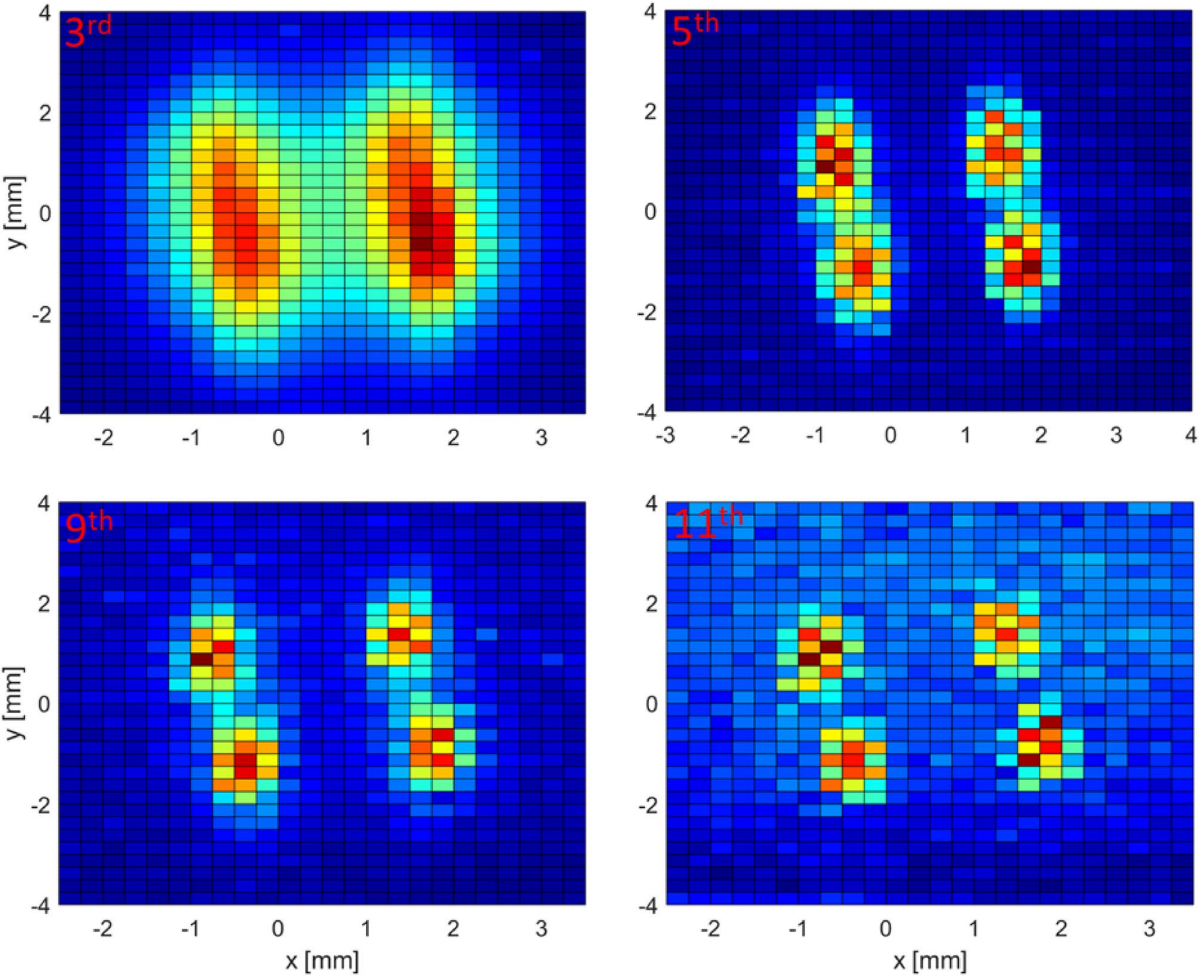
2D-MPI images of undiluted (5 mg/ml of Fe) of Vivotrax tracers in a glass phantom. Vivotrax shows a more gradual harmonic dependence of resolution than 70 nm Synomag.

To demonstrate the existence of a harmonic PSF, we acquired MPI images reconstructed using individual harmonic amplitudes by means of (1) the spectrum of the digitized time signal and (2) the lock-in amplifier demodulated signal. Using both approaches, the apparent dependence of resolution on harmonic number was observed for two different MNP samples. Here, a glass phantom with four channels was used as a sample holder for 70 nm Synomag (Fig. 7) and for Vivotrax (Fig. 8). These two widely used MNP commercial systems have significant differences in their magnetic properties but both exhibit improved spatial resolution with increasing harmonic number. Although the crystallite diameters for Vivotrax and Synomag are both about 5 nm, Vivotrax shows non-uniform aggregation, but Synomag is a “nanoflower” with well-defined cluster geometry^53^. They exhibit differences in their MPS signatures, AC susceptibility, and PSF widths. Figure 7a shows a strong resolution dependence on harmonic number, as the four wells become more spatially resolved with increasing harmonic number. To quantitatively analyze the resolution dependence, an acrylic phantom with two sample wells spaced by 0.5 mm (edge-to-edge) was machined and imaged in 1D (Fig. 7b). The spatial resolution was estimated by applying the Houston criterion^54^ to the fitted FWHM of the measured profile, and the result is plotted in Fig. 7c. The spatial resolution displays an exponential improvement with increasing harmonic number and saturates at higher harmonics. A similar analysis was done for Vivotrax tracers (Fig. 8) with an observed exponential-like dependence of resolution on harmonic number.

#### Harmonic PSF theory and experiment

To derive the fundamental origin of the harmonic PSF, or the spatial variation of the PSF with respect to harmonic number, we start by describing the 1D MPI signal of a spatial distribution $\rho (x)$ of identical particles by:

2 $$\begin{aligned} s(t)=\int _{-\infty }^{\infty }\rho (x)s_{part}(x,t)dx, \end{aligned}$$

where $s_{part}(x,t)$ is the signal from a single particle, given by:^45,52^

3 $$\begin{aligned} s_{part}(x,t)=-\varepsilon \frac{d}{dt}m_{part}\left( H_\textit{Eff}(x,t)\right) . \end{aligned}$$

This is the voltage response for the case of an inductive receive coil where $m_{part}\left( H_\textit{Eff}(x,t)\right)$ is the particle magnetic moment component in the direction of the applied field and $\varepsilon$ is the coil sensitivity. In the case of periodic excitation with an effective magnetic field $H_{\textit{Eff}}(x,t)$, Eq. (3) can be decomposed as a Fourier series:

4 $$\begin{aligned} s_{part}(t) = \sum _{n=1}^{\infty } {S_n(x)}e^{in\omega _o t}, \end{aligned}$$

where $S_n(x)$ is the spatially-dependent Fourier component and $\omega _o=2\pi f_o$ is the angular frequency of the drive field.

The effective field $H_\textit{Eff}$ is a combination of a spatially varying gradient field $H_S(x)$, with gradient G, and a time-varying excitation field $H_D(t)$, with frequency $f_o=1/T$ and amplitude *A*, such that $H_{\textit{Eff}}(x,t)=H_S(x)-H_D(t)=Gx-A\cos (\omega _o t)$. The Fourier coefficients are then given by:

5 $$\begin{aligned} S_n(x)= & {} -\frac{1}{T}\int _{-T/2}^{T/2}s_{part}(x,t)e^{-in\omega _o t}dt \nonumber \\= & {} -\frac{\varepsilon }{T}\int _{-T/2}^{T/2}m'_{part}(H_{\textit{Eff}}(x,t)) \frac{dH_{\textit{Eff}}}{dt}e^{-in\omega _o t}dt, \end{aligned}$$

where $m'_{part}(H_{\textit{Eff}})$ is the derivative of $m_{part}$ with respect to its argument. Along the drive trajectory, we can use the relation $t(H_D)=\mp \frac{1}{\omega _o}\arccos \left( \frac{H_D}{A}\right)$ to parameterize by field in the integral of Eq. (5). Arccos is only defined on the range $[0,\pi ]$, so the first half period can be identified with negative values for time and the second half with positive values. Following the development by Rahmer:^2^

6 $$\begin{aligned} S_n= & {} \frac{\varepsilon }{T}[\int _{-A}^{A}\left( m'_{part}(H_S-H_D\right) e^{in\arccos (H_D/A)}dH_D +\int _{A}^{-A}(m'_{part}(H_S-H_D)e^{-in\arccos (H_D/A)}dH_D \nonumber \\= & {} \frac{2i\varepsilon }{T}\int _{-A}^{A}\left( m'_{part}(H_S-H_D\right) \sin \left[ n\arccos \left( H_D/A\right) \right] dH_D. \end{aligned}$$

If we set the condition that

7 $$\begin{aligned} \sin (n\arccos (H_D/A))= 0~\textrm{for}~|H_D/A|>1, \end{aligned}$$

then the limits of integration in Eq. (6) can be set to infinity. Recalling that $H_S(x)=Gx$, we arrive at the following form to describe the spatial dependence of the Fourier coefficients, $S_n(x)$:

8 $$\begin{aligned} S_n(x)= & {} 2i\varepsilon f_o m'_{part}\left( H_S\right) *\sin \left[ n\arccos \left( H_s/A)\right) \right] \nonumber \\= & {} 2i\varepsilon f_o m'_{part}\left( Gx\right) *\sin \left[ n\arccos \left( Gx/A)\right) \right] . \end{aligned}$$

Equation (7), which closely resembles the Chebyshev polynomials that are truncated for $|x|>A/G$, is significant because these polynomials form a complete orthogonal basis which can describe any MPI harmonic image^2,52^.

**Figure 9:**
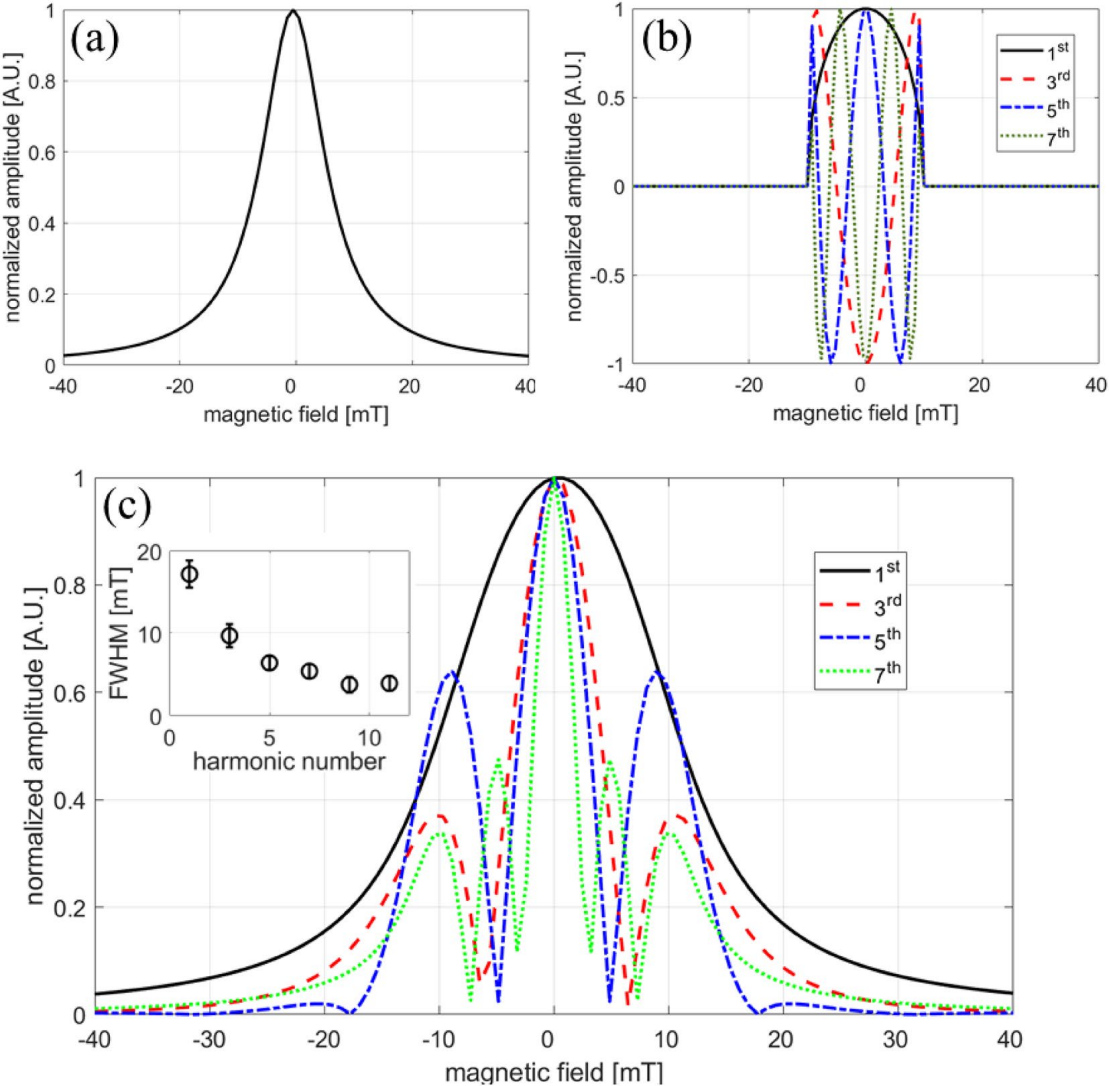
Calculation of the harmonic PSF for the 1st to 7th harmonics. (**a**) Simulated PSF using a Lorentzian function with a 13 mT FWHM. (**b**) Spatial dependence of harmonic components based on Chebyshev polynomials. (**c**) Computed harmonic PSF using Eq. 8. The inset shows the fitted FWHM using a Lorentzian function for each harmonic from 1st to 11th.

A numerical calculation of the analytical form of Eq. (8) is shown in Fig. 9 for a nanoparticle with a Langevin response and PSF FWHM of 13 mT, typical of commercial Vivotrax samples. In the Langevin case, the magnetic moment component in the direction of the applied field is:

9 $$\begin{aligned} \vec {m}_{part}(\vec {H})=M_sV_{part}\mathcal {L}(\xi \vec {H})\frac{\vec {H}}{||\vec {H}||},~\textrm{with}~\xi =\frac{\mu _0 m}{k_B T}. \end{aligned}$$

Here, $\mu _0$ is the vacuum permeability, *m* the magnitude of the particle magnetic moment. The moment is the saturation magnetization, $M_s$, multiplied by the volume of the magnetic core, and $\mathcal {L}$ is the Langevin function. In Fig. 9a, the PSF is approximated as a Lorentzian function^45^. Figure 9b shows the components of the Chebyshev polynomials corresponding to the particle harmonics with the condition set by Eq. (7). The final result calculated using Eq. (8) is presented in Fig. 9c and shows the spatial variation of the PSF for the 1st to 7th harmonics. Finally, the explicit temperature dependence of each individual harmonic PSF can be accounted for by using the analytical form for the derivative of the Langevin function^45^ through the argument $\xi$ from Eq. 9:

10 $$\begin{aligned} S_n(x)\propto & {} 2if_o \left( \frac{1}{\left( \xi Gx\right) ^2}-\frac{1}{\sinh ^2\left( \xi Gx\right) } \right) *\sin \left[ n\arccos \left( Gx/A)\right) \right] . \end{aligned}$$

**Figure 10:**
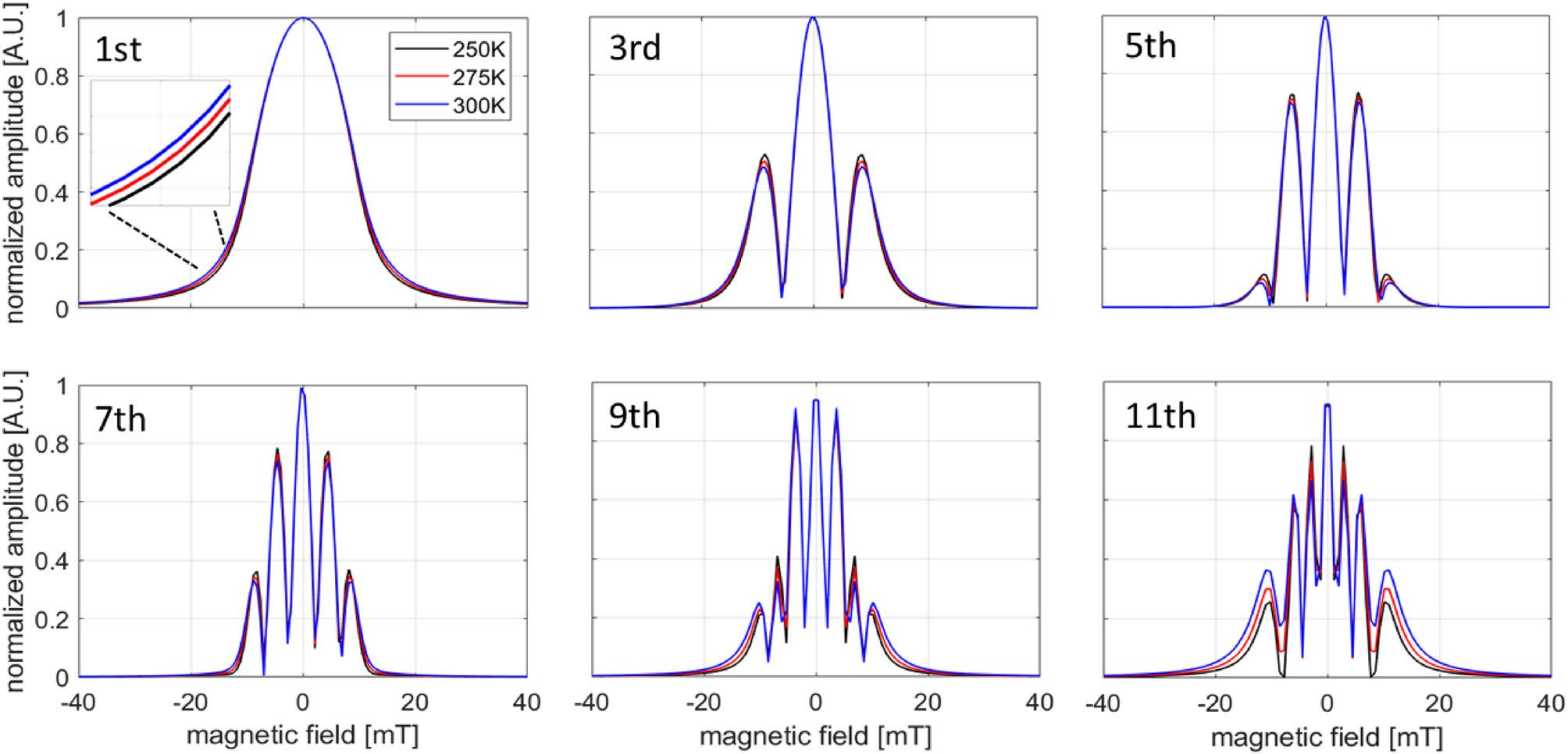
Simulated temperature-dependence of the harmonic PSF for the 1st to 11th harmonics for three temperatures (250, 275, 300 K) using Eq. (10).

A numerical calculation of the temperature-dependence for each harmonic PSF using Eq. (10) is displayed in Fig. 10. Here, a small relative change in the shape of the PSF from 250 K to 300 K is consistent with the measured data for the 7th and 9th harmonic in Fig. 4. In the scenario of (1) a low amplitude excitation magnetic field ($< 10$ mT)^46^ and (2) slow ($\approx$ 10 Hz) electromagnetic shifting or mechanical translation for image rastering, the Langevin model provides a reasonable approximation for the magnetization response because relaxation effects are minor, as evident in the minimal asymmetry observed in the negative and positive scanning of the PSF (Fig. 5c). In our PSF measurement and MPI image acquisition, the relatively smaller amplitude, but higher frequency excitation field traverses a smaller portion of the M vs H curve compared to the 8-fold larger bias field, which dominates the FFP shifting and the shape of the PSF.

According to the previous analysis, the MPI signal can be represented by a Fourier series, whose components define the harmonic PSF. To compare with the predicted form of these harmonics, we experimentally measure the individual harmonic PSF components directly by applying a slow varying (10 Hz) bias field (triangle waveform) at 80 mT peak-to-peak superimposed on the excitation field, similar to the MPS-type procedure by Croft et al.^46^. Different from previous PSF reconstruction procedures that include all harmonics, we resolved the individual harmonic PSFs by two distinct digital processing methods: (1) short-time Fourier transform (STFT) of the digitized time response signal and (2) time-swept lock-in detection.

For method (1) using the time signal *f*(*t*) from the digitizer, the STFT is implemented by first binning the digitized data in defined Hamming window segments *w*(*t*). At each time shift *t*, a new window $w(t'-t)$ is applied and the FT is calculated of $f(t')w(t'-t)$:

11 $$\begin{aligned} \hat{f}(t,u) = \int _{-\infty }^{\infty } f(t')w(t'-t)e^{-i2{\pi }t'u}dt' \end{aligned}$$

This procedure produces a spectrogram, or a plot of the Fourier spectrum at each time segment window. With the bias field turned on, the harmonic spectrum will only appear when the bias field amplitude is near zero, that is when the MNP are not fully saturated. For method (2), the harmonic PSF is obtained with the lock-in by implementing a time-domain acquisition of the output at the specified demodulation frequency ($1f_0$, $3f_0$, $5f_0$, etc.) to resolve the time-swept PSF at each harmonic number.

**Figure 11:**
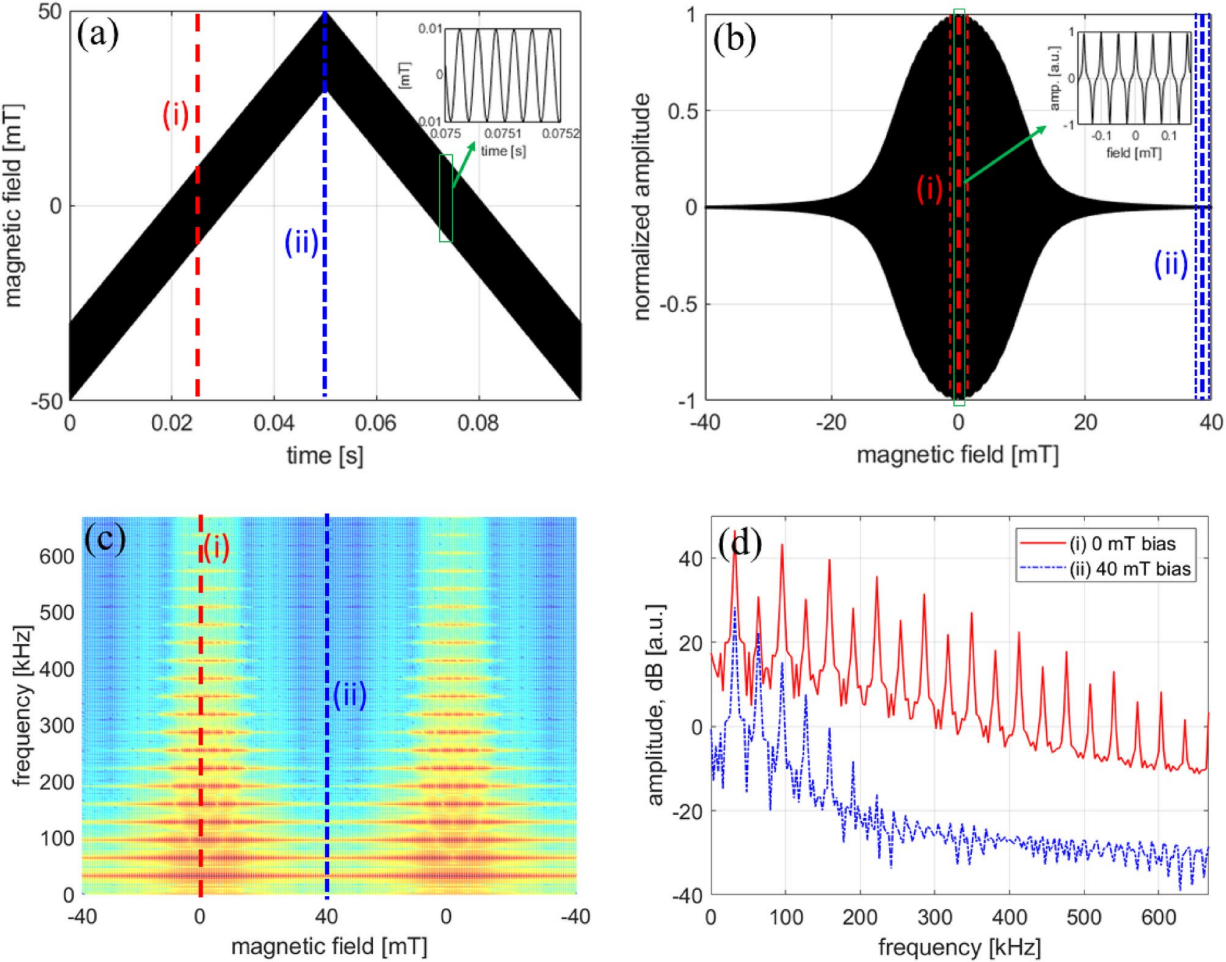
Short-time Fourier transform (STFT) procedure for obtaining the harmonic PSF at excitation and bias field frequencies $f_0 = 31.75$ kHz and $f_{bias} = 10$ Hz, respectively. (**a**) The applied field (sum of excitation and bias) as a function of time. The inset shows the $f_0$ excitation field. The red line (i) denotes the time when the bias field is 0 mT, and the blue line (ii) the time when the bias field is at its maximum of 40 mT. (**b**) The magnetization response at position (i) at the local maximum (thick red line) with the integration time window (thin red lines) and (ii) at the the local minimum (thick blue line) with the integration time window (thin blue lines) used for calculating the STFT with Eq. 2. Note that the x-axis is now converted to the magnetic field of $f_{bias}$. The inset shows the zoomed-in magnetization response at position (i). (**c**) Spectrogram of the MNP response signal using a triangular shaped window function that results in a 50 % overlap in the integration domain. The red and blue lines correspond to the time positions (i) and (ii), respectively. (**d**) Fourier spectra for the two different time positions (i) and (ii).

**Figure 12:**
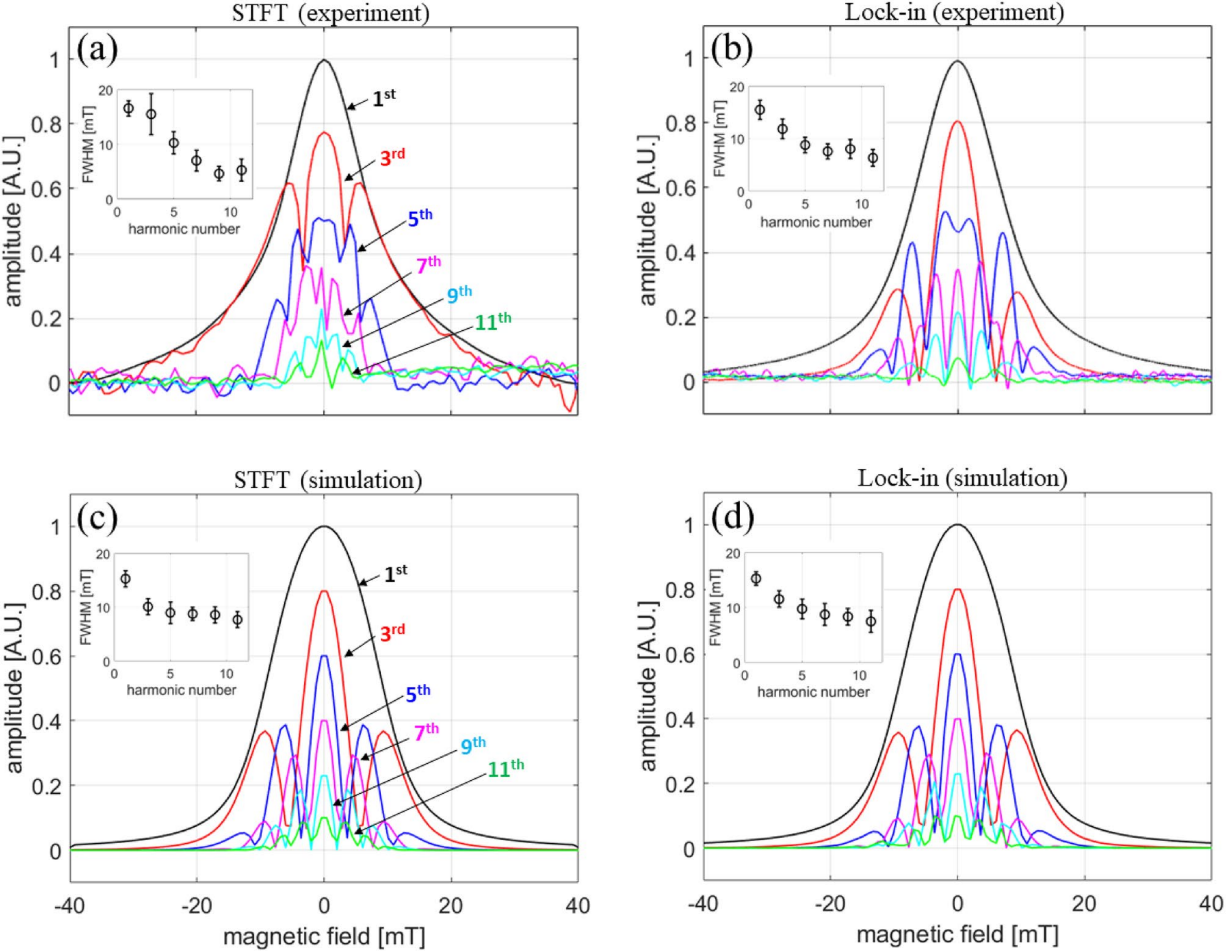
Harmonic PSF measurement of Vivotrax using a digitizer (**a**, **c**) and lock-in amplifier (**b**, **d**) at excitation frequency $f_0$ = 31.75 kHz. (**a**, **b**) and (**c**, **d**) are the measurements and simulations, respectively. For each panel, the harmonic amplitudes are scaled to 1 for the maximum value so the different panels can be easily compared. The insets shows the fitted FWHM using a Lorentzian function for each harmonic PSF.

Figure 11 is a step-by-step graphical layout of the STFT procedure with detailed descriptions in the figure caption. Harmonic PSF data taken with the digitizer (method 1, STFT) and lock-in amplifier (method 2) are plotted in Fig. 12a,b. The general shape of the PSF obtained using the two independent approaches display qualitative agreement for each harmonic, and provides experimental justification for the origin of the harmonic dependence of PSF. By inspection, it can be seen that the harmonic PSF displays a decreasing FWHM (higher spatial resolution) with increasing harmonic number, and the FWHM also converges to an asymptotic value. The insets show the fitted FWHM using a Lorentzian function for each harmonic PSF. This trend explains our imaging results for nanoparticle samples in Figs. 7 and 8, and provides experimental confirmation of the fundamental harmonic frequency to spatial frequency correspondence general to MPI, analogous to the *k*-space to image-space transformation in MRI^55^.

#### Simulations of harmonic PSF experiments

To obtain additional validation and insight into the origin of the experimentally observed spatial resolution dependence on harmonic number, we performed the following direct simulation study, taking into account the experimental parameters of our MPS-type harmonic PSF measurements. The magnetic moment of a single magnetic nanoparticle is modeled using the Langevin function, and the PSF is calculated as the time-derivative of the Langevin function^45^. Finally, the harmonic dependence of the PSF is calculated numerically for both the STFT and lock-in methods using simulated bias and excitation fields, and including information such as the STFT window width and lock-in integration time constant. Measured and simulated harmonic PSFs presented in Figs. 12a,b and c,d, respectively, show that the PSF FWHM, defined by the broad envelope at each harmonic, is narrower with increasing harmonic number, with nearly an exponential-like improvement in resolution^56^. The general shape of the harmonic PSF shows qualitative agreement with the calculated PSF shown in Fig. 9. More importantly, the simulations provide intuition for the origin of the narrower PSF for higher harmonics: as the FFP location moves further from the particle location, the signal amplitude of the higher harmonics decreases faster than that of the lower harmonics. In other words, as the FFP position moves away from the particle location, the magnetization response becomes more linear.

**Figure 13:**
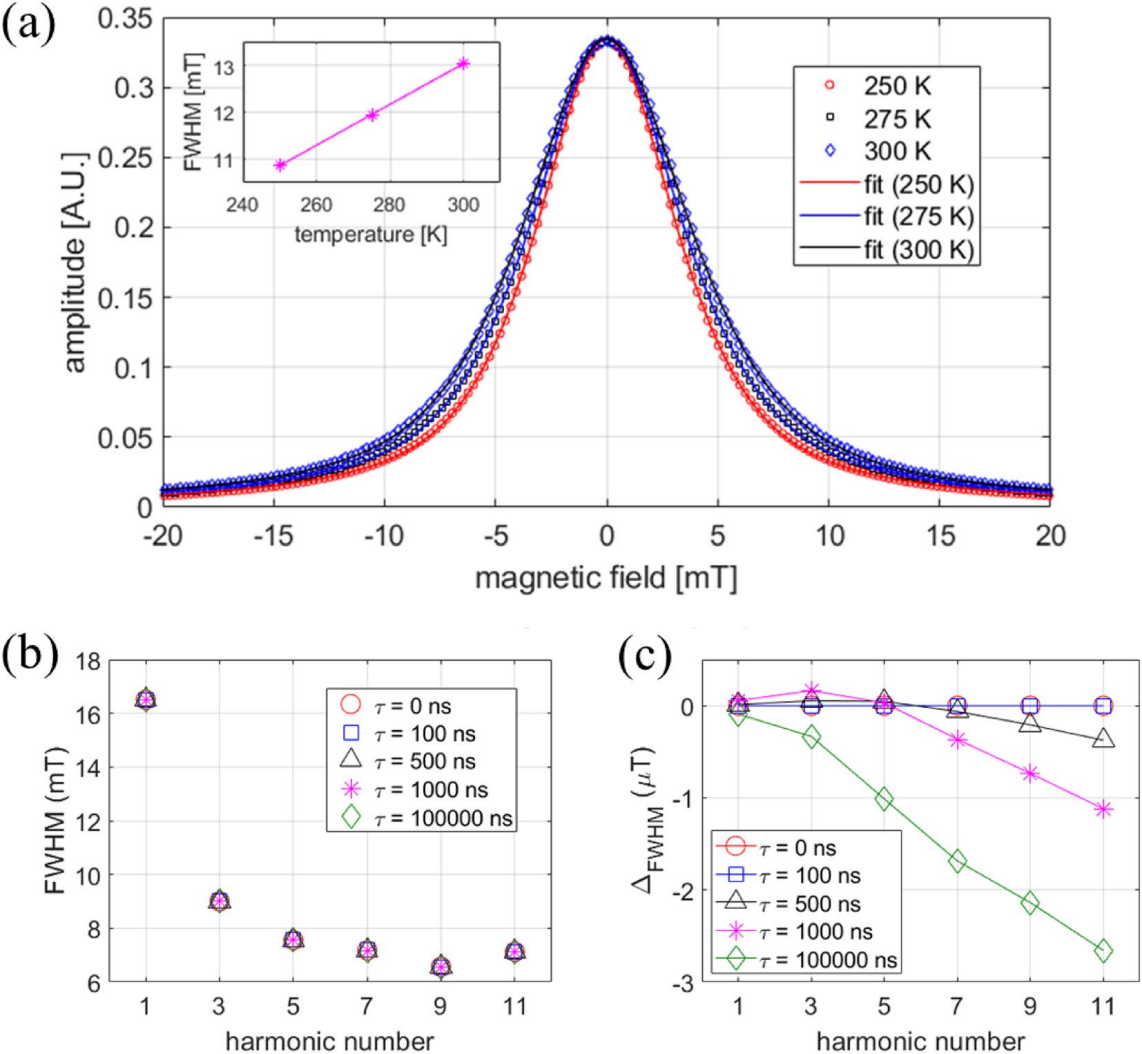
Simulations of the temperature dependence of PSF. (**a**) Langevin model: PSF for Vivotrax at three different temperatures, along with the fits using a Voigt function. The inset shows the fitted FWHM with a slope of 0.043 mT/K. (**b**) Langevin model with relaxation: FWHM of harmonic PSF (1st to 11th) for Vivotrax different relaxation times (temperature). (**c**) $\Delta _{FWHM}$ is the difference in the computed harmonic PSF FWHM with and without relaxation effects.

We also simulated the temperature dependence of the full PSF to validate the observations in Fig. 5. Note that due to the explicit temperature dependence of $\xi$ in Eq. (9), the magnetization of a single particle shows a corresponding temperature dependence, even if the saturation magnetization was set to be constant. Figure 13a shows the simulated temperature dependence of the PSF over the range 250 K to 300 K, in which only a modest change in the PSF FWHM with temperature (0.043 mT/K) was observed, in good agreement with the Vivotrax data from Fig. 5. Despite its generality, the Langevin function does not account for relaxation effects that may impact the magnetization response in MPI^51,57,58^. To account for relaxation, the Langevin response in Eq. (9) was modified to include a relaxation kernel^51,59^:

12 $$\begin{aligned} \vec {m}_{r}(\vec {t})=\vec {m}_{part}(\vec {t})*[\frac{1}{\tau }e^{(-t/\tau )}u(t)]. \end{aligned}$$

Here, $\tau$ is the effective relaxation time constant and *u*(*t*) is the Heaviside function. Despite the small impact of relaxation effects on our measurements, both Brownian and Néel relaxation time constants are affected by temperature^58,60^ and may introduce additional changes to the harmonic PSF. To quantify the impact of temperature, we used the magnetization model with relaxation (Eq. (12)) and computed the harmonic PSF with varying relaxation time constants ($\tau$ = 100 ns to 100 $\mu s$) expected within our measurement temperature range (Supplementary Information, SI). Relaxation has a negligible impact on the harmonic PSF FWHM (Fig. 13b,c), but reduces the harmonic PSF’s amplitude at increasing harmonic number (SI). The latter is consistent with other reports of relaxation effects decreasing the signal amplitude^46,61^.

## Discussion

Towards the goal of advancing temperature measurement and thermal imaging with magnetic nanoparticles, this work comprised experimental and theoretical studies on the harmonic and temperature dependence of MPI and analyses of their fundamental origin. To capture small temperature variations of the particle response with improved sensitivity, we implemented a multi-harmonic lock-in detection scheme well-tailored for the harmonic analysis approach to temperature determination. Analysis of individual harmonics in MPI revealed an apparent dependence of imaging resolution with harmonic number—the harmonic PSF. For the harmonic PSF, we have shown agreement between the analytical form, numerical simulations of experiments, and experimental results using both STFT and lock-in methods. Harmonic PSF width decreases as harmonic number increases, consistent with theory, simulations, and imaging spatial resolution in our MPI scanner measurements. Our experimental validation of the harmonic PSF establishes a direct connection between the imaging spatial resolution and harmonic measurement for MPI, which is required for understanding and predicting thermal imaging resolution using the harmonic measurement approach. This analysis of the harmonic PSF is the first step towards understanding the thermal spatial resolution. A more direct and systematic characterization of spatial resolution with temperature variations, or a temperature PSF using isolated harmonics and harmonic ratios, requires (1) a thermal gradient phantom and (2) extension of the harmonic PSF theory to the “temperature PSF” to validate measurements of a thermal gradient. This would also allow for quantification of temperature measurement resolution due to image reconstruction artifacts, e.g. from gridding errors due to particles experiencing non-zero magnetic field in the vicinity of the FFP. These studies are currently underway and beyond the scope of this work. Finally, this work also validates the correspondence of parallel multi-harmonic lock-in and digitizer acquisition for MPI imaging and thermometry, with the former displaying superior noise performance and being straightforward to implement. Further improvements in detection sensitivity will require developments of both detection and lock-in electronics that are noise-matched to the receive chain and operate at high frequencies.

## Methods

Similar to our temperature-tunable AC magnetometers^48,62^, the MNP sample holder is machined out of thermally conductive Shapal ceramic. The temperature of the sample is tuned by water-cooling with a recirculating chiller, and the temperature is measured by two 100 ohm platinum resistance thermometers. An additional water-cooling line is used to control the temperature of the excitation solenoid coil to ensure thermal stability over long acquisition times. A CAD drawing of the MPI instrument is presented in Fig. 1a. The selection field (gradient field: 20 T/m in x-axis, 10 T/m in y-xis and z-axis) is generated from a pair of permanent magnets (diameter = 50 mm, height = 50 mm, N52 grade NdFeB) separated by $\approx$ 30 mm to 40 mm (bore size) to produce the field-free-point (FFP). The FFP is fixed in our MPI imager, and spatial scanning is achieved by mechanical translation of a 3D stage in which the sample holder and excitation coils are mounted. This image acquisition scheme relies on spatial mechanical step-scanning at a maximum rate of $\approx 5$ Hz, which makes it more similar to the x-space method^12,13,52,63^ that uses low frequency ($\approx$ 10 Hz) shift coils rather than the Lissajous pattern-based electromagnetic shifting with three orthogonal fields near 25 kHz^1,7,39^.

The transmit chain is a resonant circuit at the excitation frequency ${f}_0$ = 31.75 kHz with a second order low-pass filter to attenuate (50 dB) the higher frequency feedthrough signals. Magnetization response from AC excitation (10 $\normalsize mT_p$) is recorded with an inductive receive coil, wound as a first-order gradiometer in series with an additional $\approx 30$ dB notch filter at the excitation frequency. This output is directly connected to a SR560 pre-amplifier, whose output is connected in series to an impedance matched, cascaded notch filter stage with $\approx 60$ dB of attenuation at ${f}_0$ and $\approx 40$ dB at $2 {f}_0$. The filtered magnetization response is directed to either a digitizer (GaGe, CSE161G2, 16 bit, 1 GSa/s) or lock-in amplifier (Zurich, HF2LI, 50 MHz) to measure the harmonic amplitudes for image reconstruction. For the digitizer, the harmonic amplitudes are determined from the Fourier transform of the time domain signal. The multi-frequency capability of the HF2LI lock-in amplifier allows the recording of six independent harmonics simultaneously in a parallel fashion.

### Supplementary Information

Supplementary Information.

## Data Availability

The datasets used and/or analysed during the current study available from the corresponding author on reasonable request.

## References

[1] Gleich B, Weizenecker J (2005). Tomographic imaging using the nonlinear response of magnetic particles. Nature.

[2] Rahmer J, Weizenecker J, Gleich B, Borgert J (2009). Signal encoding in magnetic particle imaging: Properties of the system function. BMC Med. Imaging.

[3] Goodwill PW, Konkle JJ, Saritas EU, Zheng B, Conolly SM (2012). Projection X-space magnetic particle imaging patrick. IEEE Trans. Med. Imaging.

[4] Saritas E (2013). Magnetic particle imaging (MPI) for NMR and MRI researchers. J. Magn. Reson..

[5] Vogel P (2014). Traveling wave magnetic particle imaging. IEEE Trans. Med. Imaging.

[6] Panagiotopoulos N (2015). Magnetic particle imaging: Current developments and future directions. Int. J. Nanomed..

[7] Gräfe K, von Gladiss A, Bringout G, Ahlborg M, Buzug TM (2016). 2d images recorded with a single-sided magnetic particle imaging scanner. IEEE Trans. Med. Imaging.

[8] Knopp T, Gdaniec N, Möddel M (2017). Magnetic particle imaging: From proof of principle to preclinical applications. Phys. Med. Biol..

[9] Vogel P (2019). Micro-traveling wave magnetic particle imaging - sub-millimeter resolution with optimized tracer LS-008. IEEE Trans. Magn..

[10] Talebloo N, Gudi M, Robertson N, Wang P (2020). Magnetic particle imaging: Current applications in biomedical research. J. Magn. Reson. Imaging.

[11] Han X (2020). The applications of magnetic particle imaging: From cell to body. Diagnostics (Basel).

[12] Kurt S, Muslu Y, Saritas EU (2020). Partial FOV center imaging (PCI): A robust X-space image reconstruction for magnetic particle imaging. IEEE Trans. Med. Imaging.

[13] Mason EE (2021). Concept for using magnetic particle imaging for intraoperative margin analysis in breast-conserving surgery. Sci. Rep..

[14] Tay ZW (2021). Magnetic particle imaging: An emerging modality with prospects in diagnosis, targeting and therapy of cancer. Cancers.

[15] Chandrasekharan P (2021). Non-radioactive and sensitive tracking of neutrophils towards inflammation using antibody functionalized magnetic particle imaging tracers. Nanotheranostics.

[16] Weaver JB, Rauwerdink AM, Hansen EW (2009). Magnetic nanoparticle temperature estimation. Med. Phys..

[17] Garaio E, Collantes JM, Garcia JA, Plazaola F, Sandre O (2015). Harmonic phases of the nanoparticle magnetization: An intrinsic temperature probe. Appl. Phys. Lett..

[18] Stehning C, Gleich BRJ (2016). Simultaneous magnetic particle imaging (MPI) and temperature mapping using multi-color MPI. Int. J. Magn. Part. Imaging.

[19] Wells J, Paysen H, Kosch O, Trahms L, Wiekhorst F (2018). Temperature dependence in magnetic particle imaging. AIP Adv..

[20] Zhong J, Schilling M, Ludwig F (2019). Excitation frequency dependence of temperature resolution in magnetic nanoparticle temperature imaging with a scanning magnetic particle spectrometer. J. Magn. Magn. Mater..

[21] Franke J (2016). System characterization of a highly integrated preclinical hybrid MPI-MRI scanner. IEEE Trans. Med. Imaging.

[22] Bauer LM, Situ SF, Griswold MA, Samia ACS (2016). High-performance iron oxide nanoparticles for magnetic particle imaging - guided hyperthermia (HMPI). Nanoscale.

[23] Hensley D (2017). Combining magnetic particle imaging and magnetic fluid hyperthermia in a theranostic platform. Phys. Med. Biol..

[24] Tay ZW (2018). Magnetic particle imaging-guided heating in vivo using gradient fields for arbitrary localization of magnetic hyperthermia therapy. ACS Nano.

[25] Chandrasekharan P (2020). Using magnetic particle imaging systems to localize and guide magnetic hyperthermia treatment: Tracers, hardware, and future medical applications. Theranostics.

[26] Salamon J (2020). Visualization of spatial and temporal temperature distributions with magnetic particle imaging for liver tumor ablation therapy. Sci. Rep..

[27] Weidensteiner C (2003). Real-time MR temperature mapping of rabbit liver in vivo during thermal ablation. Magn. Reson. Med..

[28] Perälä J (2014). MRI-guided laser ablation of neuroendocrine tumor hepatic metastases. Acta Radiol. Short Rep..

[29] Rahmer J, Halkola A, Gleich B, Schmale I, Borgert J (2015). First experimental evidence of the feasibility of multi-color magnetic particle imaging. Phys. Med. Biol..

[30] Pantke D, Holle N, Mogarkar A, Straub M, Schulz V (2019). Multifrequency magnetic particle imaging enabled by a combined passive and active drive field feed-through compensation approach. Med. Phys..

[31] Rauwerdink AM, Hansen EW, Weaver JB (2009). Nanoparticle temperature estimation in combined ac and dc magnetic fields. Phys. Med. Biol..

[32] Quelhas, K. N., Henn, M.-A., Farias, R. C., Tew, W. L. & Woods, S. I. Parallel 3d temperature image reconstruction using multi-color magnetic particle imaging (mpi). *AIP Conference Proceedings* (2023, In Press).

[33] Zhong J, Dieckhoff J, Schilling M, Ludwig F (2016). Influence of static magnetic field strength on the temperature resolution of a magnetic nanoparticle thermometer. J. Appl. Phys..

[34] Draack S, Viereck T, Kuhlmann C, Schilling M, Ludwig F (2017). Temperature-dependent MPS measurements. Int. J. Magn. Part. Imaging.

[35] Draack S (2019). Determination of dominating relaxation mechanisms from temperature-dependent Magnetic Particle Spectroscopy measurements. J. Magn. Magn. Mater..

[36] Janssen K-J, Zhong J, Viereck T, Schilling M, Ludwig F (2022). Quantitative temperature visualization with single harmonic-based magnetic particle imaging. J. Magn. Magn. Mater..

[37] Goodwill PW, Scott GC, Stang PP, Conolly SM (2009). Narrowband magnetic particle imaging. IEEE Trans. Med. Imaging.

[38] Graeser M, Knopp T, Grüttner M, Sattel TF, Buzug TM (2013). Analog receive signal processing for magnetic particle imaging. Med. Phys..

[39] Weizenecker J, Gleich B, Rahmer J, Dahnke H, Borgert J (2009). Three-dimensional real-time in vivo magnetic particle imaging. Phys. Med. Biol..

[40] Zheng B (2017). Optimal broadband noise matching to inductive sensors: Application to magnetic particle imaging. IEEE Trans. Biomed. Circuits Syst..

[41] Malhotra A, Schwegmann H, Schumacher J, Chen X, Buzug TM (2020). Fully differential low noise amplifier for MPI/MPS. Int. J. Magn. Part. Imaging.

[42] Mason, E. E. *Magnetic Particle Imaging for Intraoperative Breast Cancer Margin Assessment and Functional Brain Imaging*. Ph.D. thesis, Massachusetts Institute of Technology (2020).

[43] Arami H (2017). Tomographic magnetic particle imaging of cancer targeted nanoparticles. Nanoscale.

[44] Trisnanto SB, Takemura Y (2019). Modulating relaxation responses of magnetic nanotracers for submillimeter imaging. Appl. Phys. Lett..

[45] Goodwill PW, Conolly SM (2010). The x-space formulation of the magnetic particle imaging process: 1-d signal, resolution, bandwidth, snr, sar, and magnetostimulation. IEEE Trans. Med. Imaging.

[46] Croft LR (2016). Low drive field amplitude for improved image resolution in magnetic particle imaging. Med. Phys..

[47] Tay ZW (2021). Superferromagnetic nanoparticles enable order-of-magnitude resolution & sensitivity gain in magnetic particle imaging. Small Methods.

[48] Bui TQ (2022). Advanced characterization of magnetization dynamics in iron oxide magnetic nanoparticle tracers. Appl. Phys. Lett..

[49] Hankiewicz JH, Celinski Z, Stupic KF, Anderson NR, Camley RE (2016). Ferromagnetic particles as magnetic resonance imaging temperature sensors. Nat. Commun..

[50] Abel FM (2023). Thermosensitivity through exchange coupling in ferrimagnetic/antiferromagnetic nano-objects for magnetic-based thermometry. ACS Appl. Mater. Interfaces..

[51] Croft LR, Goodwill PW, Conolly SM (2012). Relaxation in x-space magnetic particle imaging. IEEE Trans. Med. Imaging.

[52] Lu K, Goodwill PW, Saritas EU, Zheng B, Conolly SM (2013). Linearity and shift invariance for quantitative magnetic particle imaging. IEEE Trans. Med. Imaging.

[53] Rietberg MT (2021). Modelling of dynamic behaviour in magnetic nanoparticles. Nanomaterials.

[54] Houston WV (1927). A compound interferometer for fine structure work. Phys. Rev..

[55] Sodickson A, Cory DG (1998). A generalized k-space formalism for treating the spatial aspects of a variety of nmr experiments. Prog. Nucl. Magn. Reson. Spectrosc..

[56] Henn M, Bui T, Woods S (2023). Investigating the harmonic dependence of MPI resolution. Int. J. Magn. Part. Imaging.

[57] Tay ZW, Hensley DW, Vreeland EC, Zheng B, Conolly SM (2017). The relaxation wall: Experimental limits to improving MPI spatial resolution by increasing nanoparticle core size. Biomed. Phys. Eng. Express.

[58] Arsalani S (2023). Temperature dependent magnetorelaxometry of magnetic nanoparticle ensembles. Phys. Med. Biol..

[59] Henn M-A, Quelhas KN, Bui TQ, Woods SI (2022). Improving model-based MPI image reconstructions: Baseline recovery, receive coil sensitivity, relaxation and uncertainty estimation. Int. J. Magn. Part. Imaging.

[60] Deissler RJ, Wu Y, Martens MA (2014). Dependence of brownian and néel relaxation times on magnetic field strength. Med. Phys..

[61] Tay ZW (2019). Pulsed excitation in magnetic particle imaging. IEEE Trans. Med. Imaging.

[62] Bui TQ, Tew WL, Woods SI (2020). Ac magnetometry with active stabilization and harmonic suppression for magnetic nanoparticle spectroscopy and thermometry. J. Appl. Phys..

[63] Goodwill PW (2012). X-space MPI: Magnetic nanoparticles for safe medical imaging. Adv. Mater..

