# Supplementary material for: Harmonic dependence of thermal magnetic particle imaging
Source: Sci Rep. 2023 Sep 22;13:15762. doi: 10.1038/s41598-023-42620-1 (PMC10516919; doi:10.1038/s41598-023-42620-1)
Supplement: Supplementary file 1 — Supplementary Information. [file 41598_2023_42620_MOESM1_ESM.pdf]

Supplementary Information (SI)

To account for temperature effects on the harmonic PSF, temperature dependent relaxation effects (i.e., effective relaxation time constants, τ_{eff}) were computed based on reported parameters (Table 1S) for Vivotrax and Synomag:

Sample	Vivotrax	70 nm Synomag
Anisotropy constant, K (J/m^3)	21000 [1]	30000 [1]
Hydrodynamic diameter (nm)	62(4) [2]	60(18) [1]
Core diameter (nm)	6.0(5) [2]	29 (9) [1]
Hydrodynamic volume, V_M (m^3)	$1.2x10^{-22}$	$1.1x10^{-22}$
Core volume, V_C (m^3)	$1.1x10^{-25}$	$1.3x10^{-23}$

Table 1S: Parameters for calculating relaxation time constants.

Using the known relations for the Néel and Brownian relaxation time constants, $\tau_N = \tau_0 * exp(KV_M/(k_BT))$ and $\tau_B = 3\eta V_H/(k_BT)$, respectively, the effective relaxation time constant, τ_{eff}, for each MNP sample was calculated by $\tau_{eff} = (\tau_N\tau_B)/(\tau_N+\tau_B)$ and presented in Table 2S.

Temperature (K)	Viscosity, η (Pa/s) [3]	τ_{eff}, s (Vivotrax)	τ_{eff}, s (70 nm Synomag)
273	0.0018	$1.88x10^{-9}$	$1.62x10^{-4}$
283	0.0013	$1.84x10^{-9}$	$1.13x10^{-4}$
293	0.001	$1.80x10^{-9}$	$8.39x10^{-5}$
303	0.0008	$1.76x10^{-9}$	$6.49x10^{-5}$
313	0.00065	$1.73x10^{-9}$	$5.10x10^{-5}$

Table 2S: Computed effective relaxation time constants.

Using Eq. 12 of the main text, the PSF over a range of relaxation time constants (100 ns to 100 μs) were calculated to account for the temperature dependence of spatial resolution over our measurement temperature range. Figures 1S-3S show the PSF swept response at three representative relaxation time constants 500 ns, 1 μs, and 5 μs, showing the impact of relaxation on the magnetization response signal.

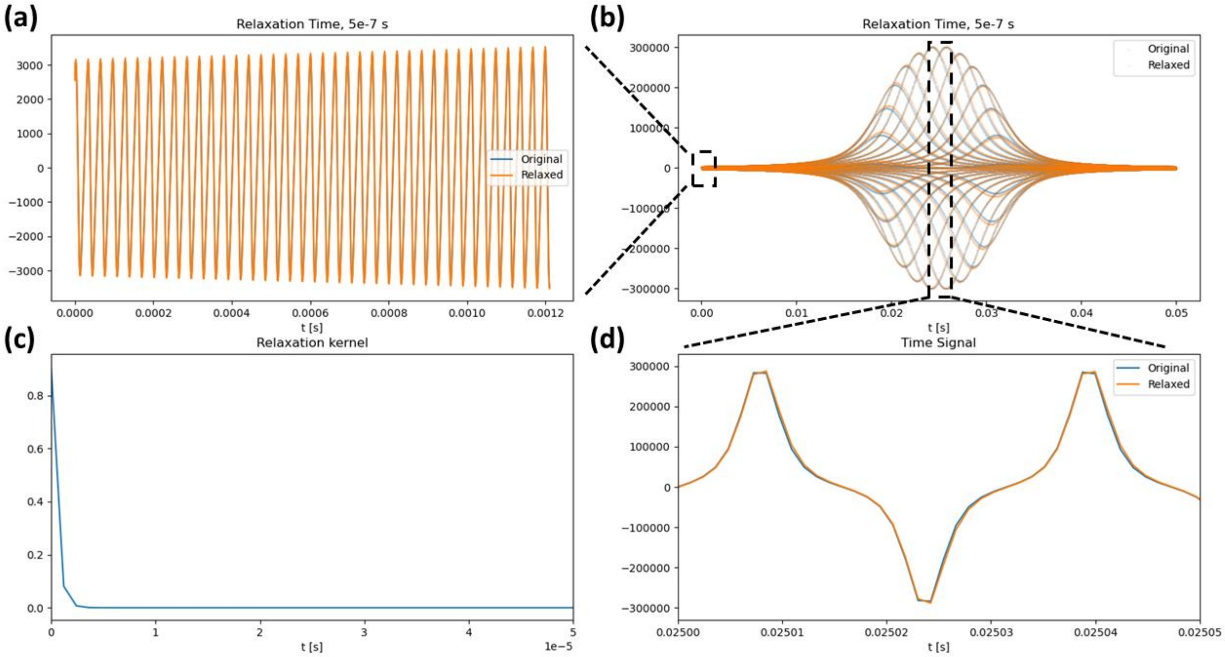

Figure 1S: τ_{eff} = 500 ns. The PSF sweep is shown in panel (b) for the magnetization model with and without relaxation. (a) and (d) show the indicated zoomed-in segments. Panel (c) is the relaxation kernel.

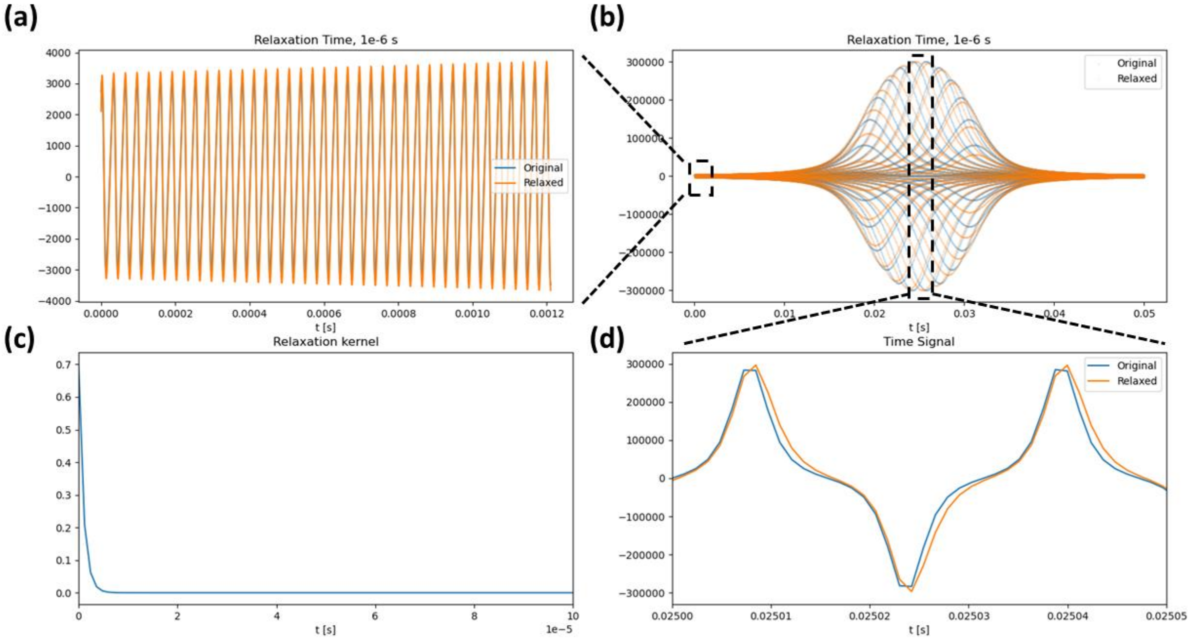

Figure 2S: τ_{eff} = 1 μs.

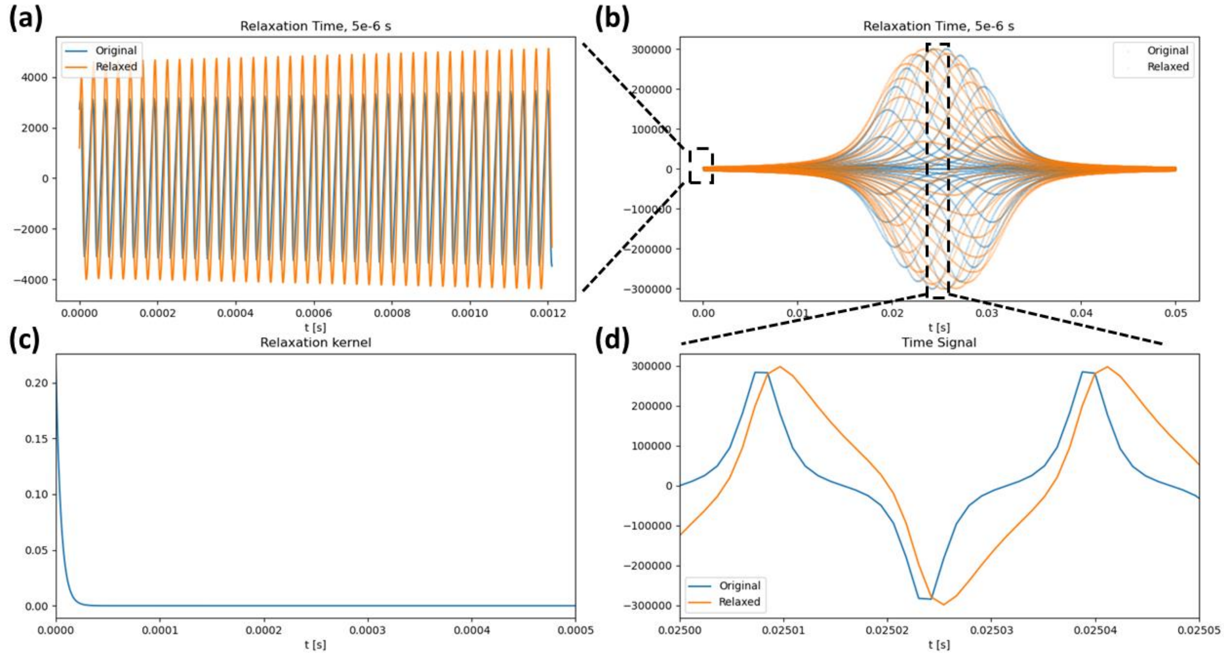

Figure 3S: τ_{eff} = 5 μs.

The harmonic PSFs in Figure 4S-6S were calculated using the STFT method for both the magnetization models with and without relaxation effects for three representative relaxation time constants.

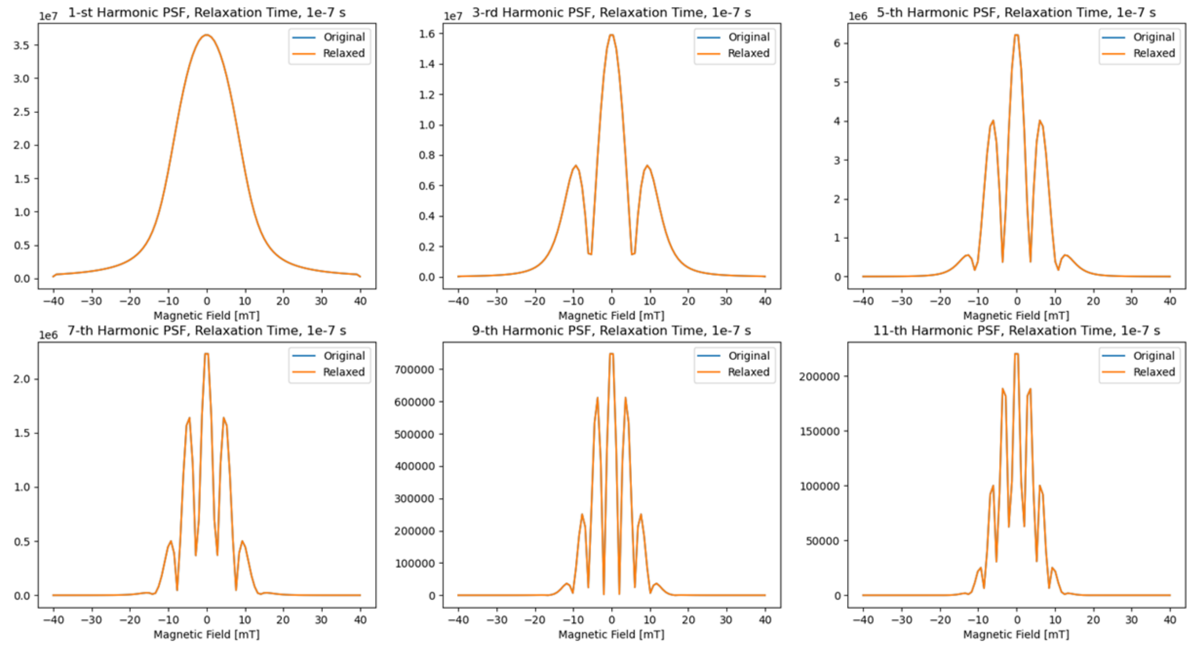

Figure 4S: τ_{eff} = 100 ns

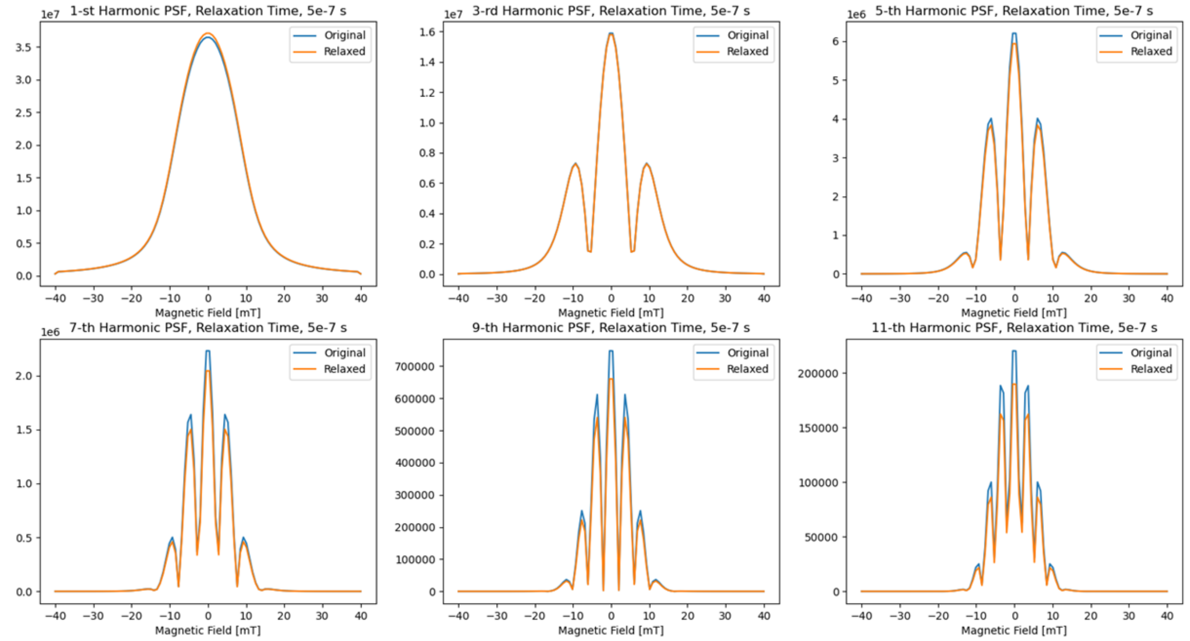

Figure 5S: τ_{eff} = 500 ns.

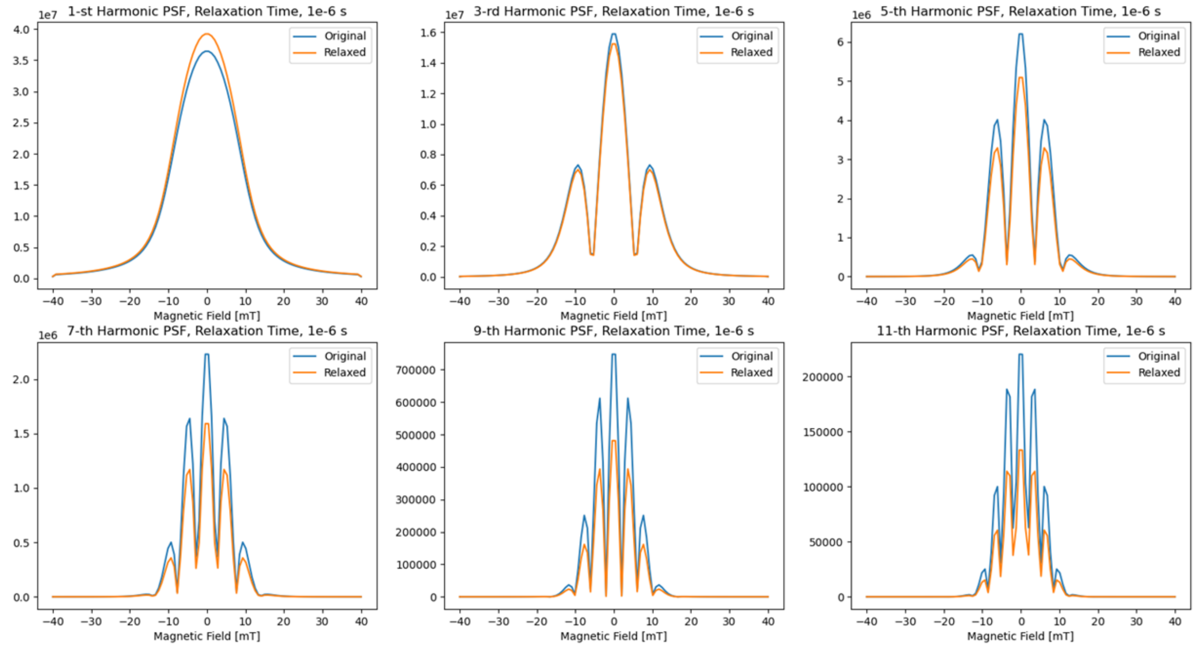

Figure 6S: τ_{eff} = 1 µs.

These results show that relaxation effects on the **shape** of the harmonic PSF are not significant, with the largest apparent change in the FWHM on the first harmonic. Two additional observations are that (1) relaxation effects mainly reduce the amplitude of the harmonic PSFs of

the higher harmonics and (2) the faster the relaxation time constant, the less impact relaxation effects have on the PSF. For time constants shorter than 100 ns (e.g., Néel dominated systems like Vivotrax), there were no observable differences on the calculated harmonic PSF with and without relaxation over our measurement range.

References

[1] Liu et al. 2021, Nanotheranostics, 5(3), 348-361. DOI: 10.7150/ntno.58548

[2] Magnetic Insight (https://www.magneticinsight.com/)

[3] https://www.engineeringtoolbox.com/water-dynamic-kinematic-viscosity-d_596.html
